# Author Correction: Digital automation of transdermal drug delivery with high spatiotemporal resolution

**DOI:** 10.1038/s41467-025-58812-4

**Published:** 2025-04-10

**Authors:** Yihang Wang, Zeka Chen, Brayden Davis, Will Lipman, Sicheng Xing, Lin Zhang, Tian Wang, Priyash Hafiz, Wanrong Xie, Zijie Yan, Zhili Huang, Juan Song, Wubin Bai

**Affiliations:** 1 Department of Applied Physical Sciences, University of North Carolina at Chapel Hill, Chapel Hill, NC 27599 USA; 2 Department of Pharmacology, University of North Carolina at Chapel Hill, Chapel Hill, NC 27599 USA; 3 UNC/NCSU Joint Department of Biomedical Engineering, Chapel Hill, NC 27599 USA; 4 Department of Psychology and Neuroscience, University of North Carolina at chapel Hill, Chapel Hill, NC 27599 USA; 5 State Key Laboratory of Medical Neurobiology, Fudan University, Shanghai, 200032 China

**Keywords:** Biomedical engineering, Electronic devices, Drug regulation, Sleep disorders, Drug delivery

Correction to: *Nature Communications* 10.1038/s41467-023-44532-0, published online 30 January 2024

In the version of the article initially published, there was an error in the Fig. 2 caption, where in the text now reading “Scale bar: 1mmm in optical images, 75 μm in SEM images,” 75 μm was originally written as “30 μm.” The Fig. 6 caption was incomplete and has been amended to cite more figure panels in the text now reading “The recording is a 3-h period from 19:00 to 22:00 in **b**–**d** and a 6-h period from 19:00-1:00 in **f**–**h**.” The last sentence of the Fig. 6 caption has been amended to include time measurement description in the text now reading “Source data are provided as a Source Data file, where time duration in **c**,**d** and **g**,**h** is counted in half-minute units.” There were errors in the Fig. 6c, g *y*-axis labels where, in the labels now reading “Time spent in each stage (min)”, the labels originally read “…(min/0.5 h),” and in the Fig. 6d, h *y*-axis labels now reading “Amount of each stage (min),” the Fig. 6d label originally read “…(min/3 h)” and the Fig. 6h label read “…(min/6 h).”

In the last sentence of the Results “Release control and electrical triggering of microneedle patch” section, “(10 V peak-to-peak signal)” has been added to the sentence now reading “The input signal’s optimal frequency is around 36 MHz (10 V peak-to-peak signal), as shown in Fig. 3f.” In the Methods section “On-demand stepwise release” section, in the text now reading “The patch was immersed in a petri dish with 20 mL of 1X DPBS at 65 °C,” the sentence originally read “…20 mL of DI water at 60 °C.” In the Methods “Finite element analysis of crevice corrosion” section, in the sentence now reading “The tip of the MN was treated as a hemisphere (50 μm in diameter),” the diameter was originally described as “100 μm.” In the Methods “Gold layer thickness analysis” section, the figure citation in the sentence now reading “Furthermore, we utilize AFM to characterize the surface roughness of the coated gold layer on the polymer substrate, as presented in Supplementary Fig. 11” originally referred to Supplementary Fig. 10. In the first sentence of the “Stereotaxic surgery (additional)” section of Methods now reading “For in vivo photometry recordings, mice were unilaterally injected with 250 nl of AAV5-CaMKII-GCaMP6f,” there was a typo in the virus name (originally reading “AAV5-CaMKIIG-CaMP6f”). The changes are made in the HTML and PDF versions of the article.

