# Supplementary material for: Digital automation of transdermal drug delivery with high spatiotemporal resolution
Source: Nat Commun. 2024 Jan 13;15:511. doi: 10.1038/s41467-023-44532-0 (PMC10787768; doi:10.1038/s41467-023-44532-0)
Supplement: Supplementary file 1 — Supplementary Information [file 41467_2023_44532_MOESM1_ESM.pdf]

Supplementary Information

Digital automation of transdermal drug delivery with high spatiotemporal resolution

Yihang Wang[1#], Zeka Chen[2#], Brayden Davis[3], Will Lipman[3], Sicheng Xing[3], Lin Zhang[1], Tian Wang[3], Priyash Hafiz[1], Wanrong Xie[1], Zijie Yan[1], Zhili Huang[4], Juan Song[2*], and Wubin Bai[1*]

[1]Department of Applied Physical Sciences, The University of North Carolina at Chapel Hill, Chapel Hill, NC, 27514, USA

[2]Department of Pharmacology, The University of North Carolina at Chapel Hill, Chapel Hill, NC, 27514, USA

[3]UNC/NCSU Joint Department of Biomedical Engineering, Chapel Hill, NC, 27514, USA

[4]State Key Laboratory of Medical Neurobiology, Fudan University, Shanghai, 200032, China

[#]These authors contributed equally: Yihang Wang, Zeka Chen

*Corresponding authors: Wubin Bai (email: wbai@unc.edu), Juan Song (email: juansong@email.unc.edu)

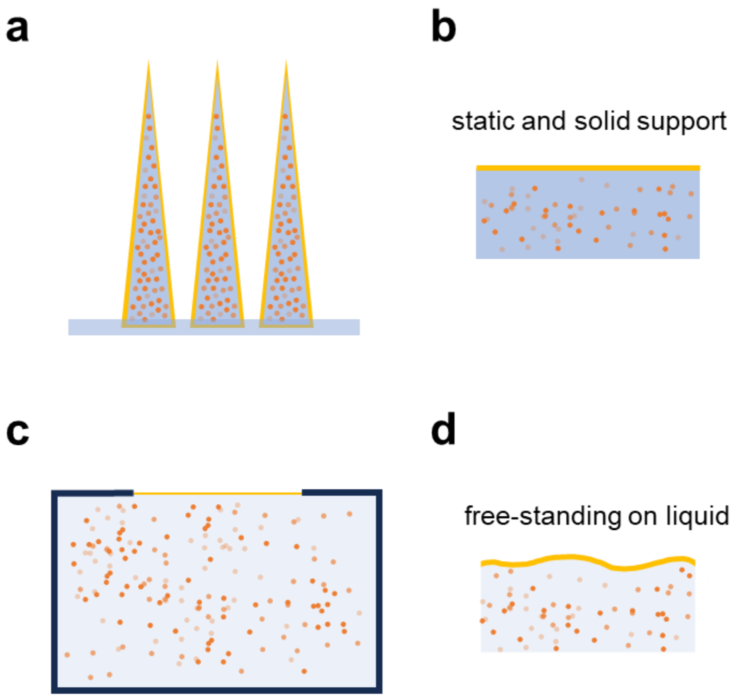

SI Figure 1. The comparison between microneedles and drug reservoirs with metallic gates. a. Schematic illustration of the drug-loaded microneedles with a layer of gold coating. **b.** The microscale illustration of the gold-PLGA interface in **a**. **c.** Schematic illustration of the drug reservoir with a liquid payload and a gate of gold membrane. **d.** The microscale illustration of the interface between the suspended gold membrane and liquid drug solution in **c**.

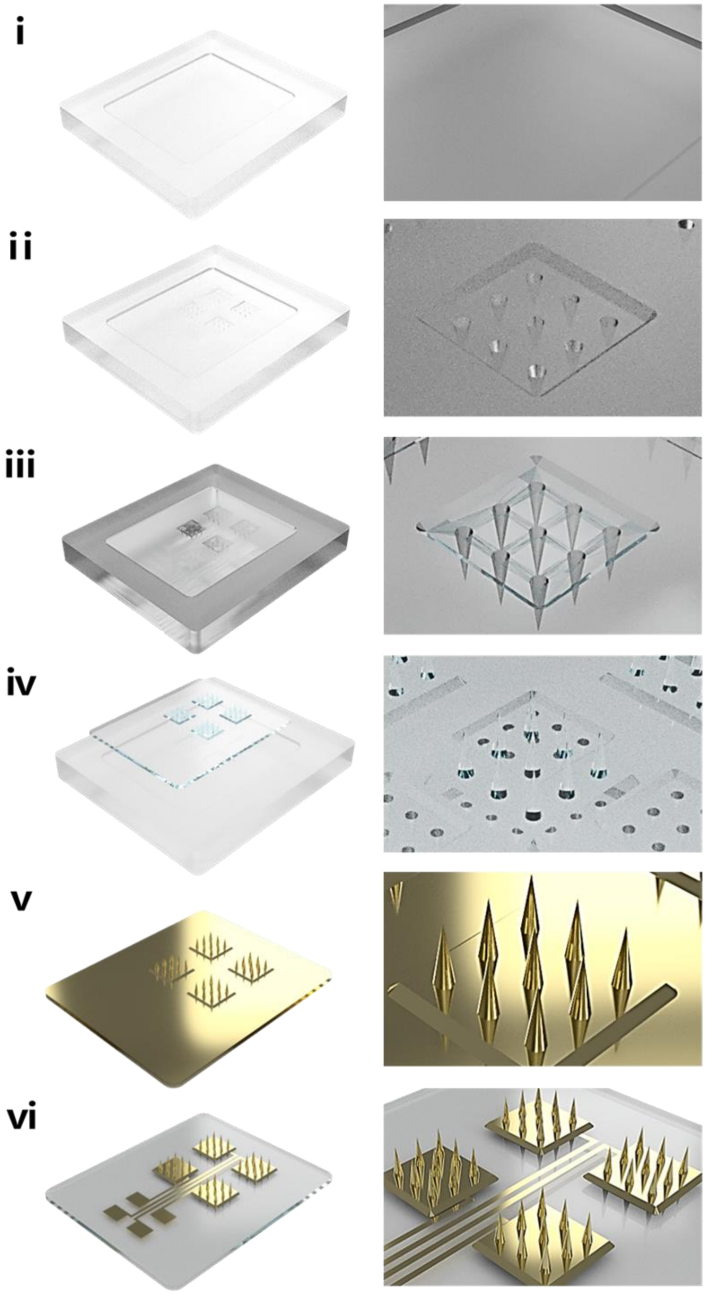

SI Figure 2. Schematic illustration of the fabrication process of SOP. i. PDMS mold curing. **ii.** UV laser ablation of MN negative molds. **iii.** PLGA mold casting of PLGA solution. **iv.** PLGA MN patch extraction. **v.** gold deposition by sputtering. **vi.** IR laser patterning of gold.

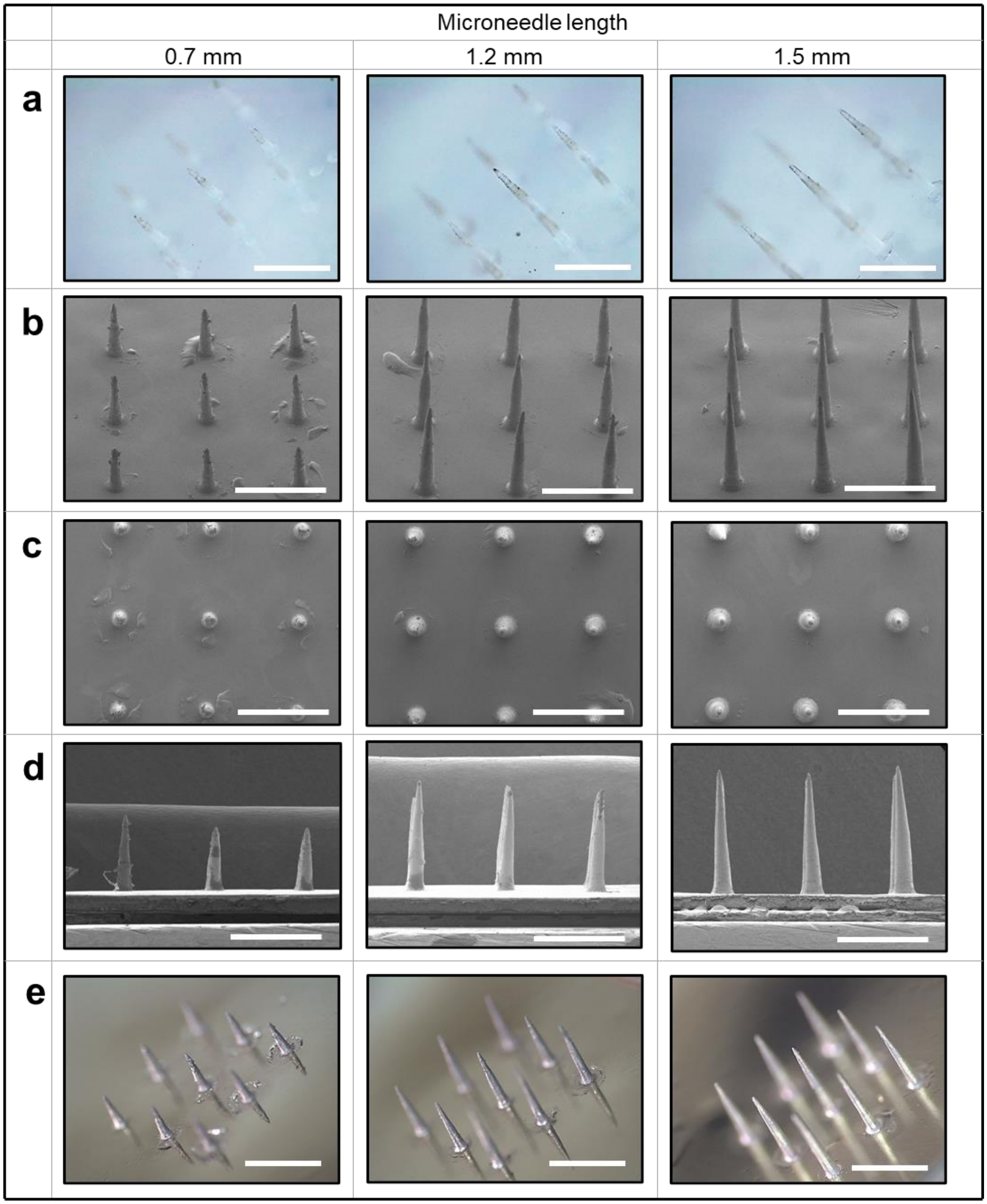

Microneedle length
0.7 mm
1.2 mm
1.5 mm
a
b
c
d
e

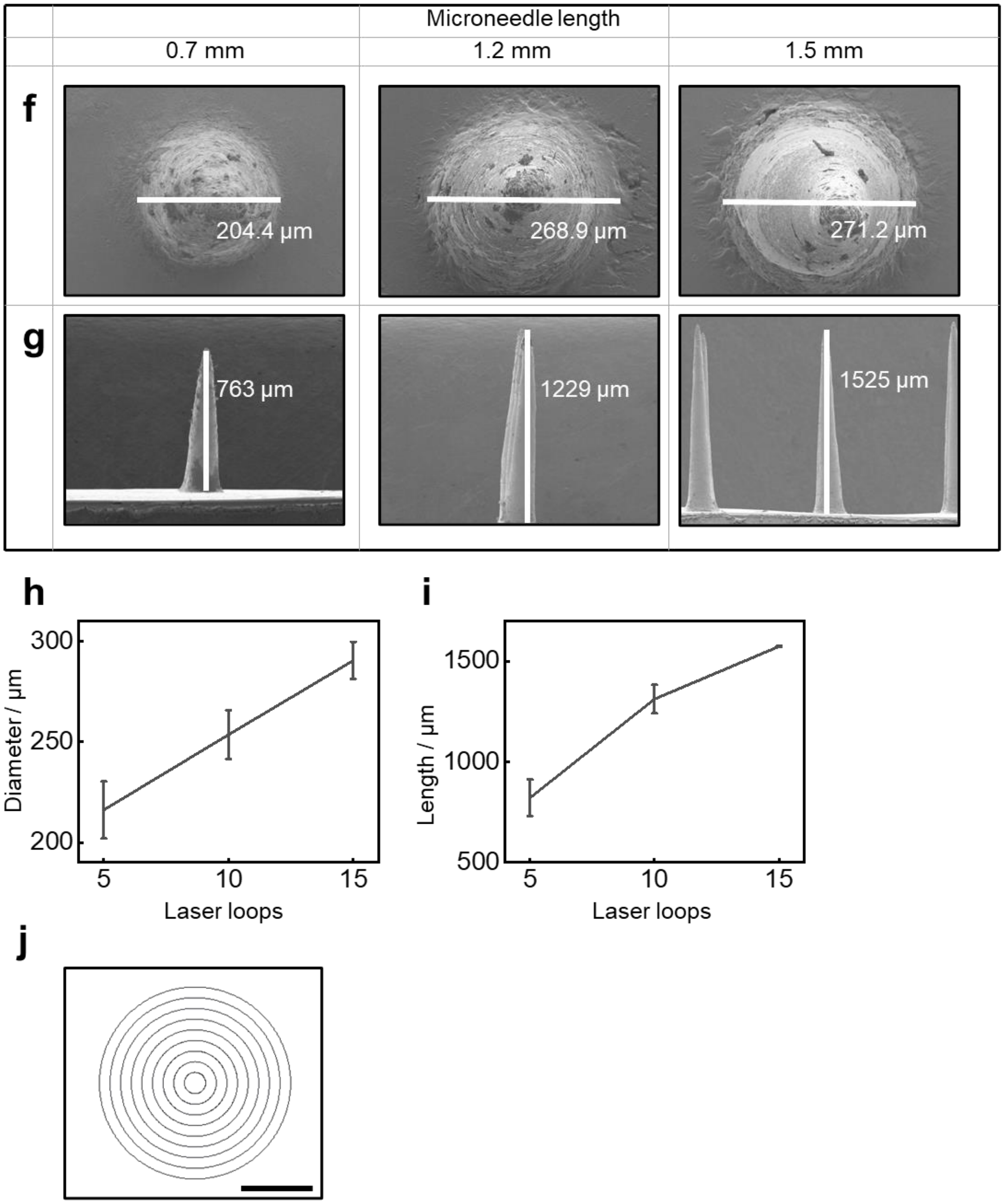

SI Figure 3. Optical and electron microscopy of microneedles of different dimensions. a. Optical images of PLGA MNs. **b.** SEM of PLGA MNs from the 45-degree perspective. **c.** SEM of PLGA MNs from the top perspective. **d.** SEM of PLGA MNs from the horizontal perspective. **e.** Optical images of gold-coated MNs. **f.** Measurement of the base diameter of MNs. **g.** Measurement of the length of MNs. **h.** Statistical analysis of the base diameter of different MNs. Data are presented as mean values +/- standard deviation of the base diameter of multiple MNs. (n=9) **i.** Statistical analysis of the length of different MNs. Data are presented as mean values +/- standard deviation of the length of multiple MNs. (n=3) **j.** The laser pattern for UV ablation of a

microneedle with a base diameter of 270 μm. Scale bar: 1 mm in **a**, **b**, **c**, **d**, **e**, 100 μm in **j**. Similar results in SI Figure 3 are obtained from 3 experiments.

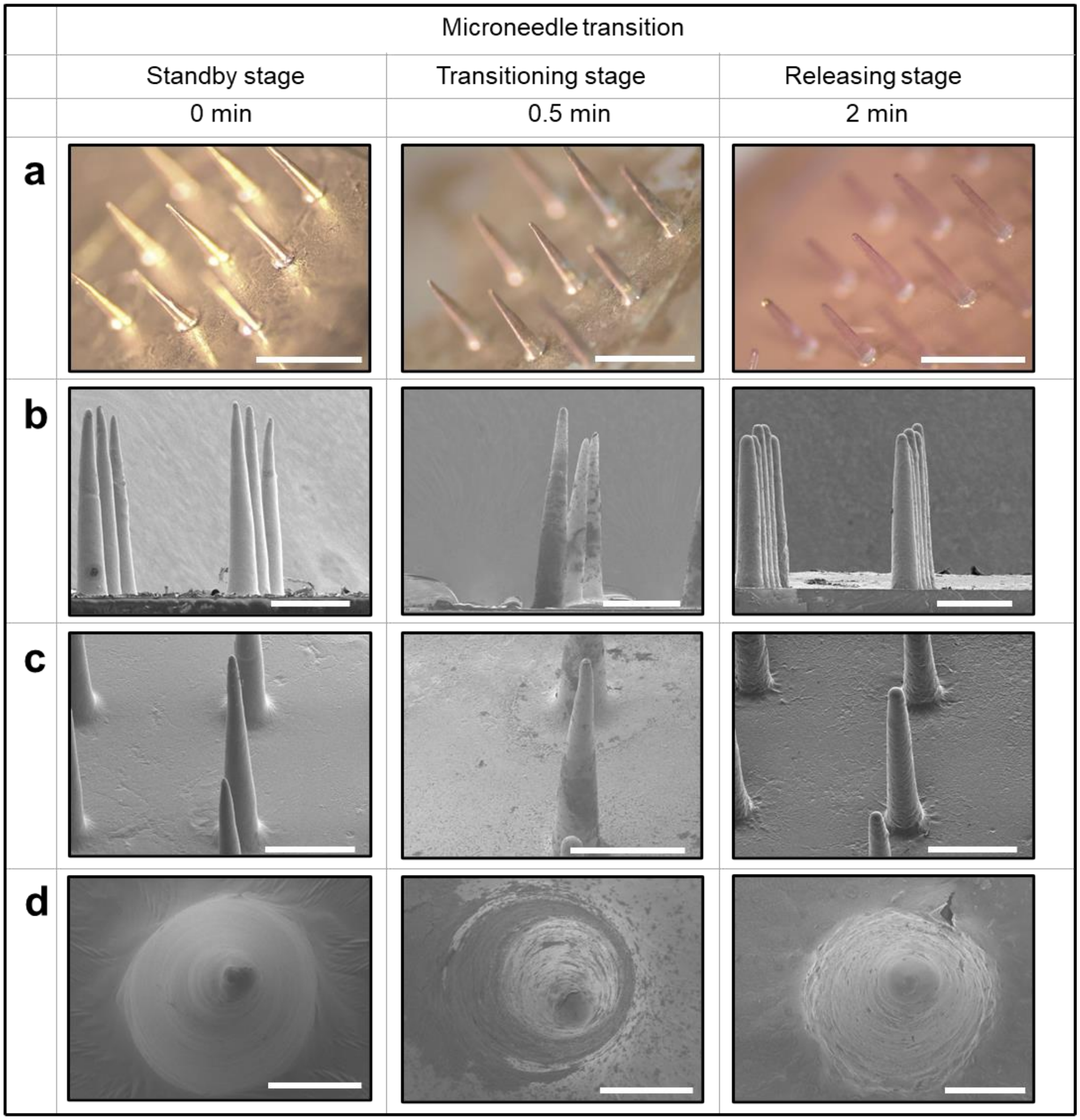

SI Figure 4. Optical and electron microscopy of microneedles at different stages of electrochemical crevice corrosion (standby stage at 0 min, transitioning stage at 0.5 min, and releasing stage at 2 min). a. Optical images of MNs from the standby stage to the releasing stage. **b.** SEM of MNs from the standby stage to the releasing stage from the horizontal perspective. **c.** SEM of MNs from the standby stage to the releasing stage from the 45-degree perspective. **d.** SEM of MNs from the standby stage to the releasing stage from the top perspective. Scale bar: 1 mm in **a**, 500 μm in **b**, **c**, 100 μm in **d**. Similar results in SI Figure 4 are obtained from 3 experiments.

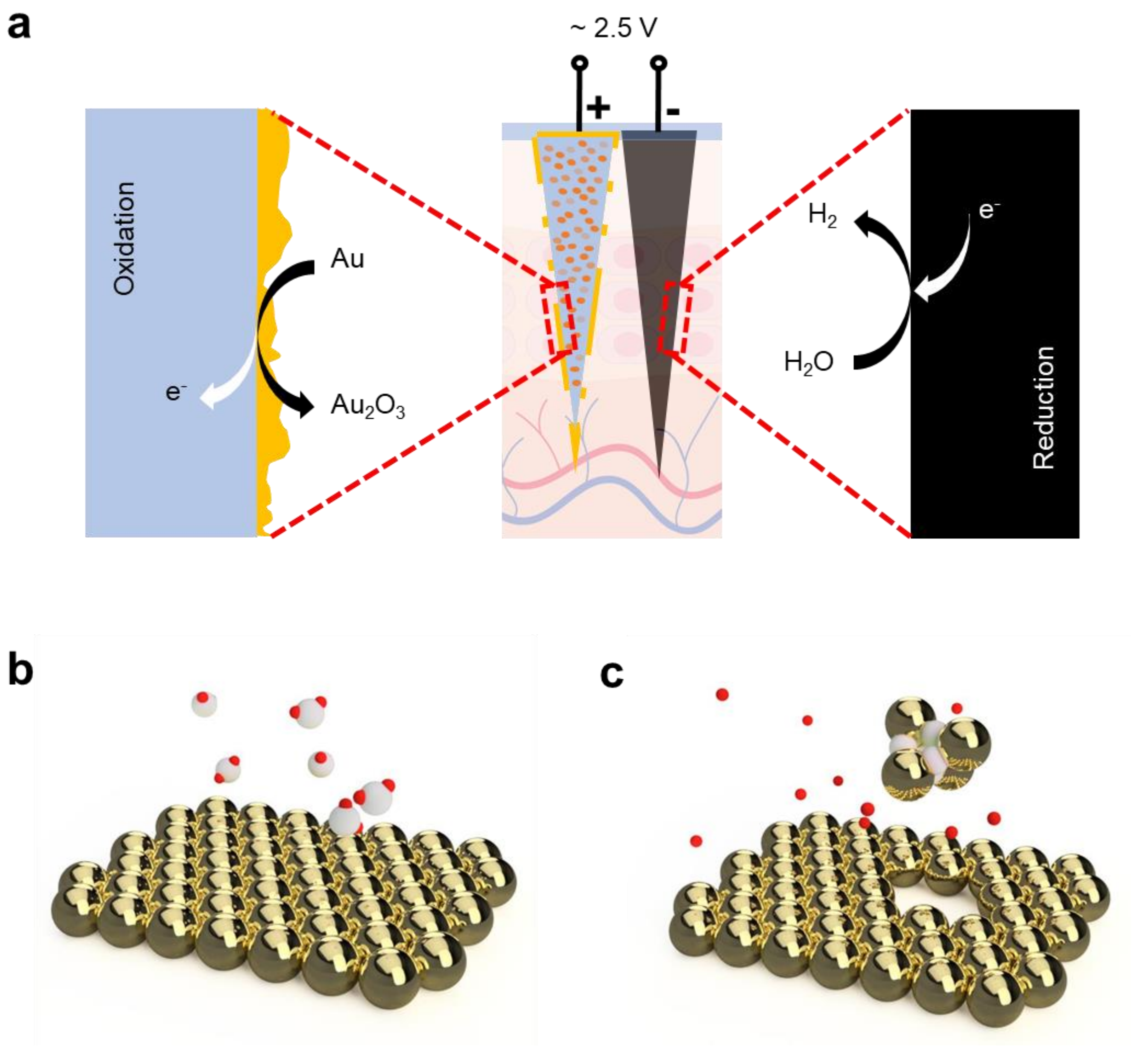

SI Figure 5. Schematic illustration of the crevice corrosion of gold in biofluids. a. Schematic illustration of anode and cathode half-reactions of the two-electrode system in SOP. **b. c.** The microscopic illustration of the anodic oxidation reaction of gold ([111] facet). **b.** Standby stage before crevice corrosion (without triggering potential applied). **c.** Transitioning stage during crevice corrosion (with triggering potential applied). Au, O, and H atoms are in golden, white, and red, respectively.

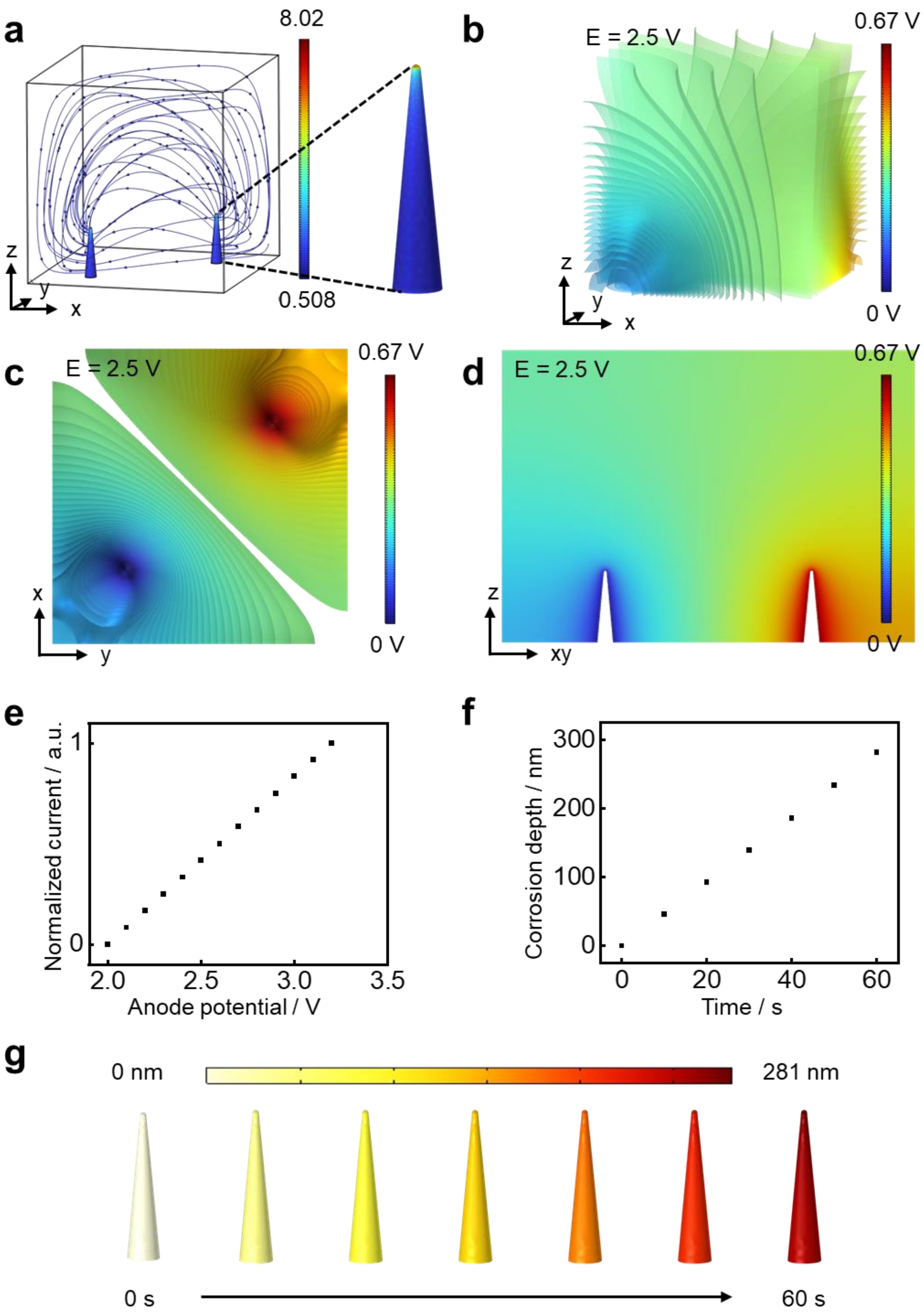

SI Figure 6. Finite element analysis of a two-microneedle model. a. The two-MN model (1.2-mm, 3.5-mm in the distance, in a 5-mm cubic space) and current distribution (a.u.) at 2.5 V. Both current distributions in the electrolyte and on the surface are visualized. **b.-d.** The iso-potential surface from different perspectives (**b.-c.**) and potential distribution on the diagonal plane (**d.**) at 2.5 V. **e.** The anodic polarization curve with the standardized current of the crevice corrosion from 2.0-3.5 V. **f.-g.** The corrosion rate of gold at 2.5 V and the corresponding visualization.

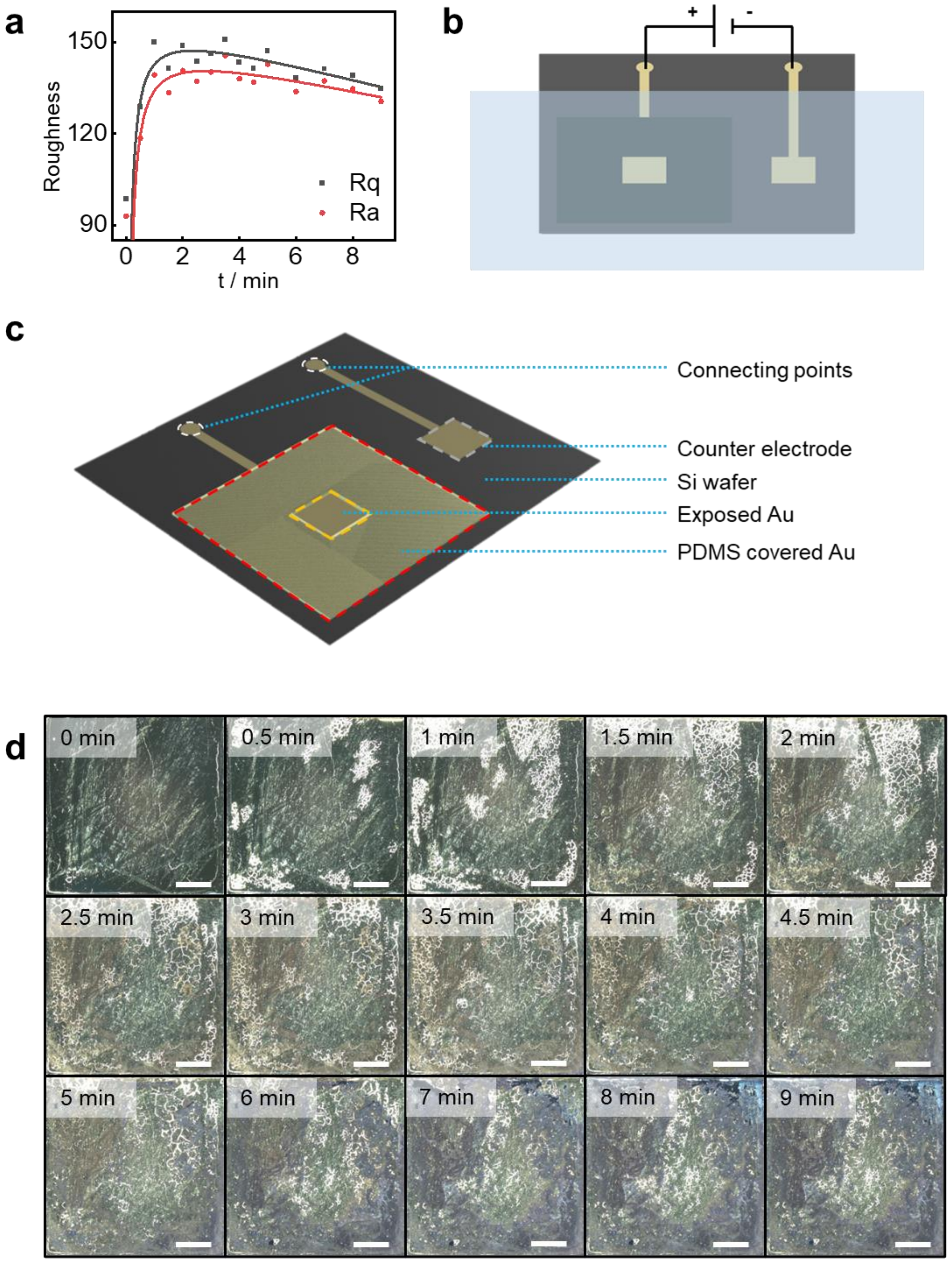

SI Figure 7. Surface profilometry of gold-on-wafer during electrochemical crevice corrosion. a. The roughness analysis of the gold surface during corrosion, with RMS roughness (R_q) in black and arithmetic roughness (R_a) in red. **b.** Schematic illustration of the experiment setup. A two-electrode system with a DC power source is used in the 1X DPBS environment. Both

the cathode and anode are 150-nm gold layers. A 2.5-V constant voltage triggers the crevice corrosion. **c.** The zoom-in view of the experimental device. Gold traces are connected to a power source by graphene tape and protected by PDMS (~ 10 μm). 1 * 1 cm^2 windows on both cathode and anode are left open to allow exposure. **d.** Optical images of the exposed gold region on the anode during crevice corrosion. The white areas are recognized as exfoliated gold. Scale bar: 2 mm. Similar results are obtained from 2 experiments.

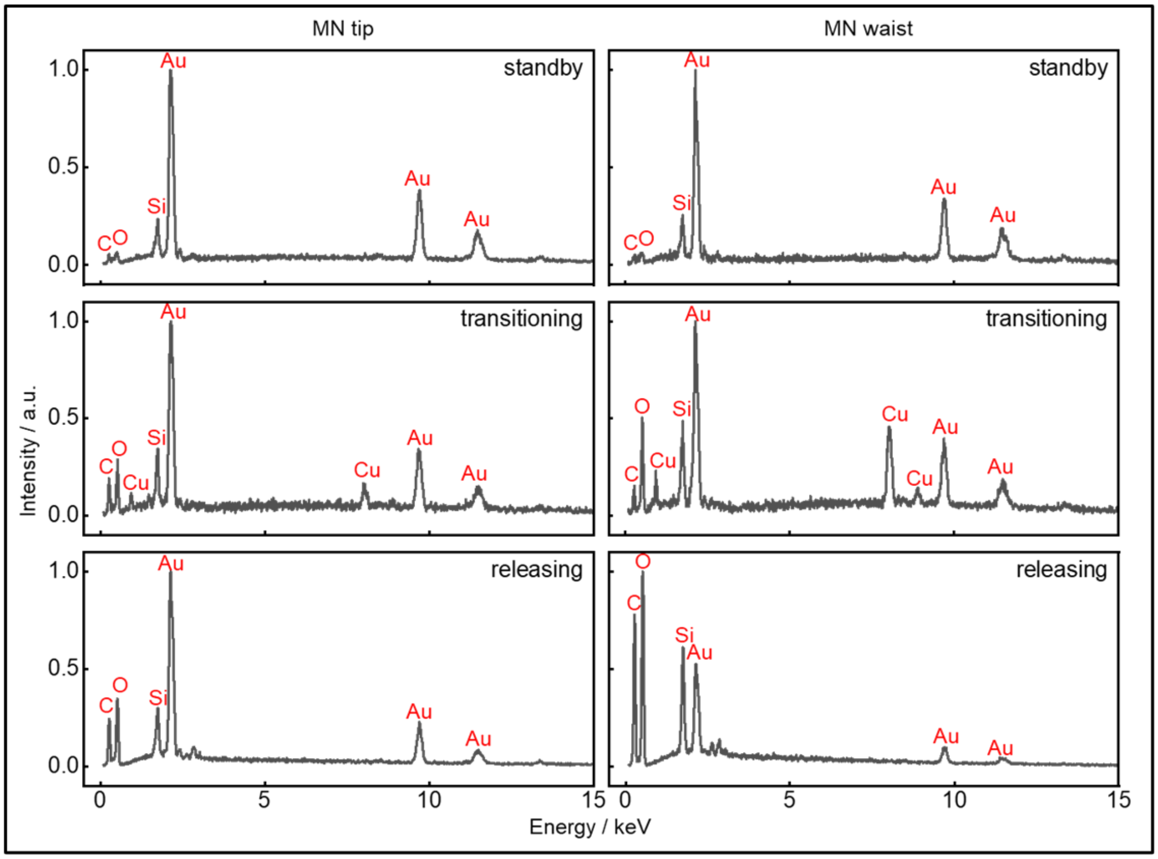

SI Figure 8. EDXS spectra of microneedles from different stages of electrochemical corrosion on two areas (the top area and the waist area). The corresponding elements of EDXS peaks are labeled in red. Stages of corrosion are labeled as standby, transitioning, and releasing, corresponding to the stages in SI Figure 4.

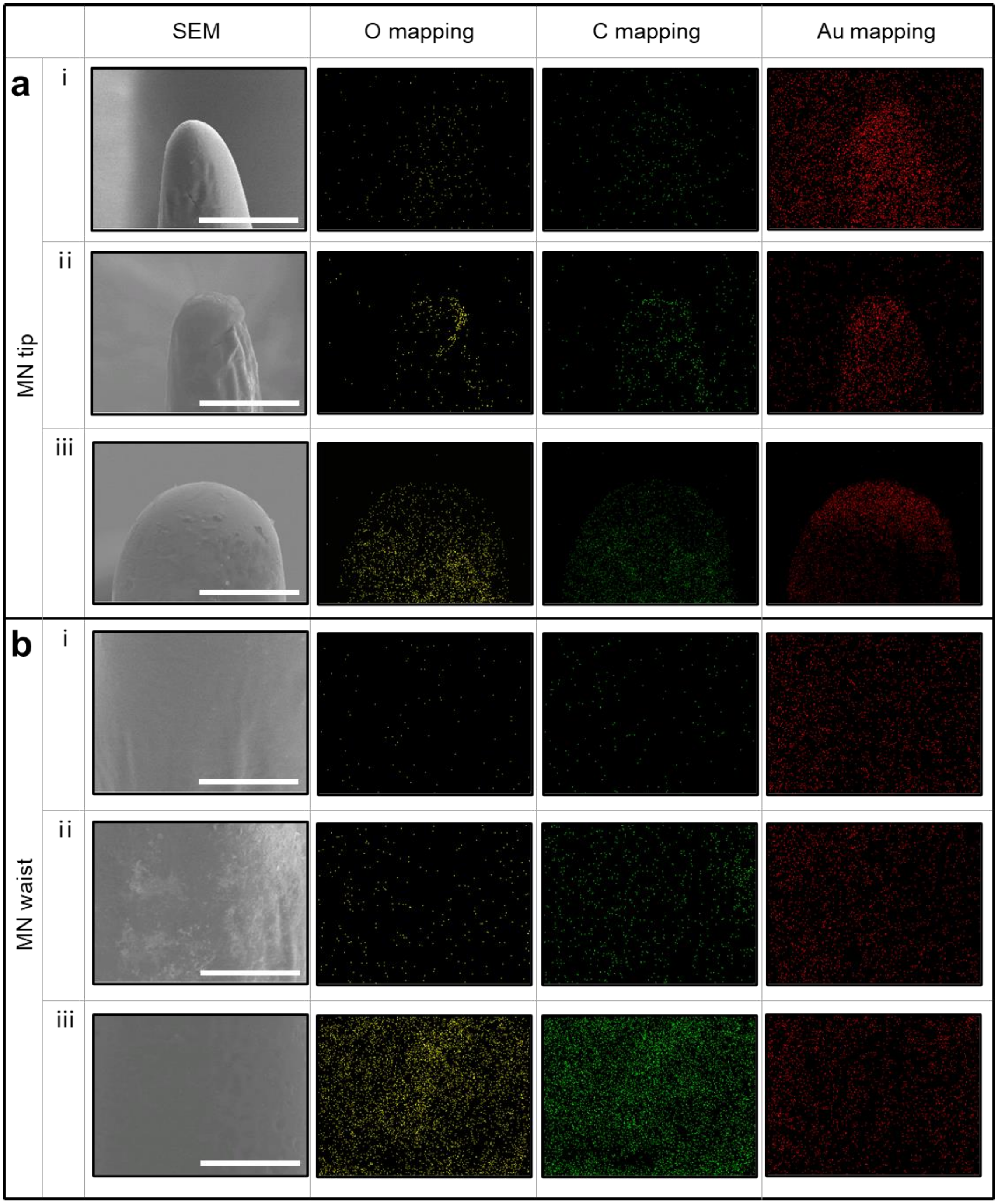

SI Figure 9. EDXS element mapping of oxygen (O), carbon (C), and gold (Au) from different stages of electrochemical crevice corrosion on two parts of the microneedle (the tip and the waist). The oxygen, carbon, and gold mappings are in yellow, green, and red, respectively. **a.** The high magnification SEM image, oxygen, and carbon mapping of the tip area of MNs from three stages: **i.** standby; **ii.** transitioning; **iii.** releasing. **b.** The high magnification SEM image, oxygen, and carbon mapping of the waist area of MNs from three stages: **i.** standby; **ii.** transitioning; **iii.** releasing. Scale bar: 60 μm.

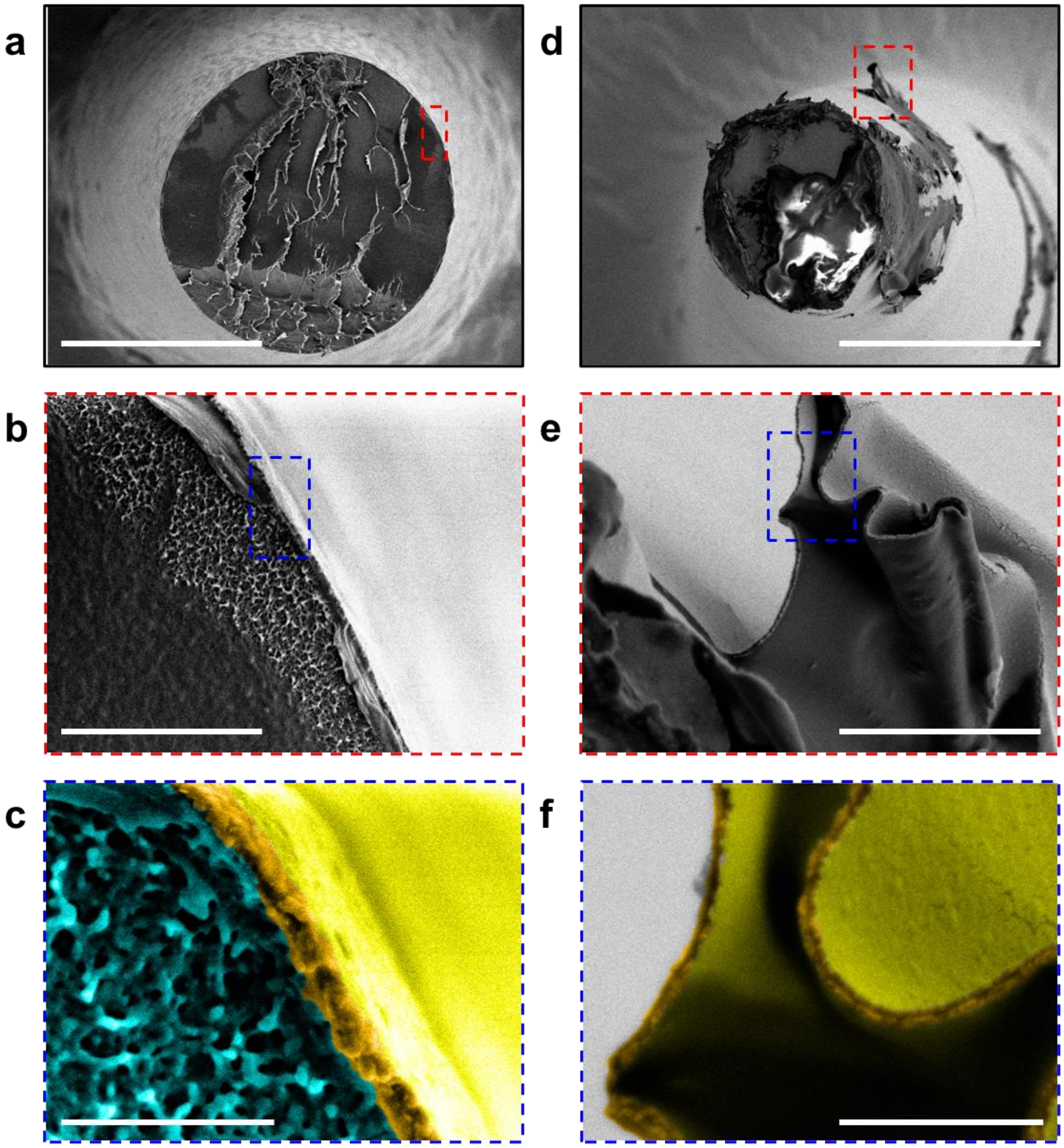

SI Figure 10. The SEM of Au film cross sections. a. b. c. The SEM images of an MN (150 nm gold coated, 1.5 mm) under various magnifications. **d. e. f.** The SEM images of a partially exfoliated gold-coated MN (100 nm gold coated, 1.5 mm) under various magnifications. The magnified areas are approximately labeled by red dash frames in **a** and **d**, and blue dash frames in **b** and **e**. Cross-sections of gold film are colored orange, gold film surfaces are colored yellow, and the PLGA cross-section is colored light blue. Scale bars: 100 µm in **a** and **d**, 5 µm in **b** and **e**, 1 µm in **c** and **f**.

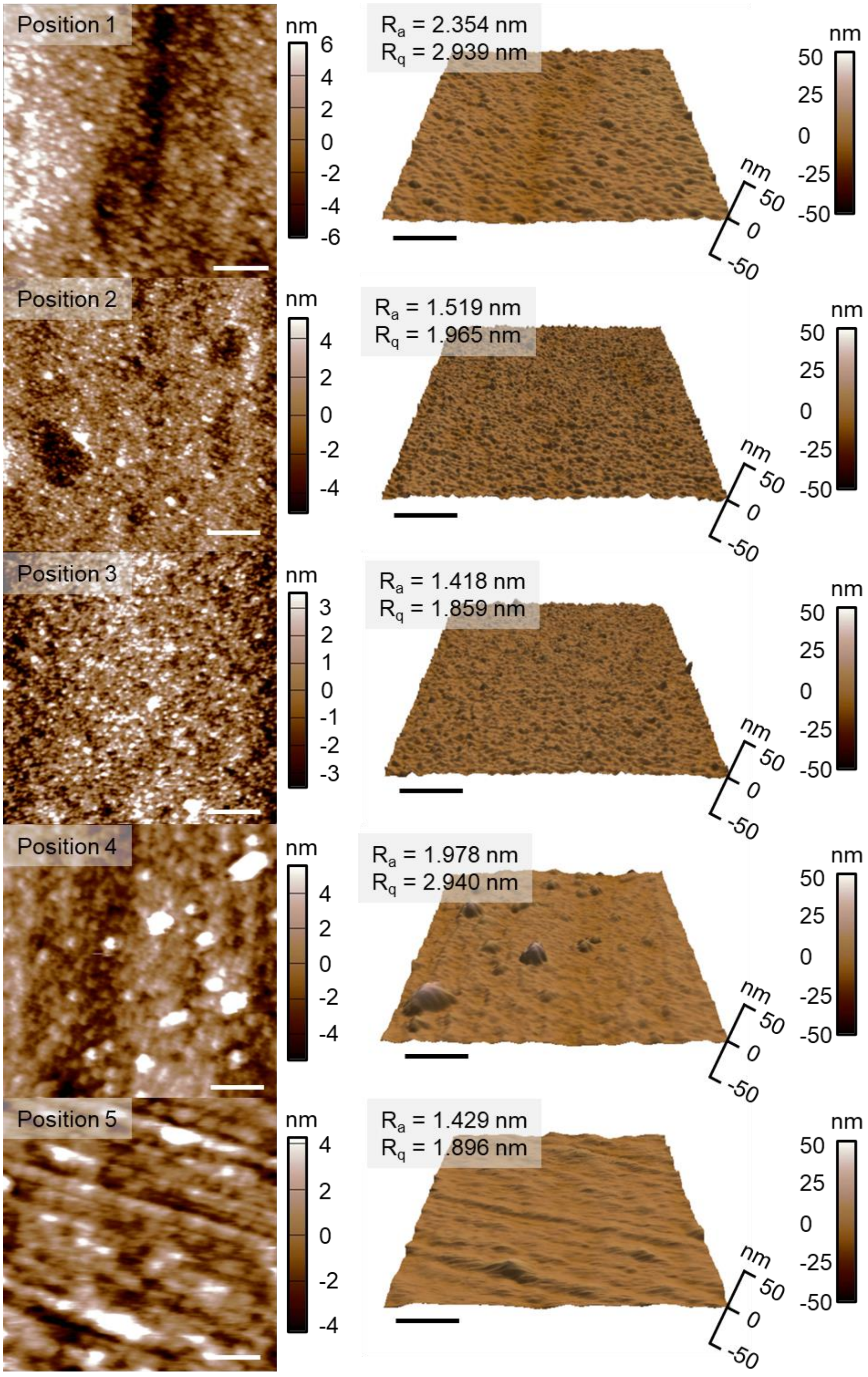

SI Figure 11. Surface profile of sputter-coated Au film on polymer substrate by AFM. Five different positions (labeled as Position 1-5) are characterized by AFM with roughness calculated. Both 2D and 3D maps are provided for each position. RMS roughness (R_q) and arithmetic roughness (R_a) are attached to the 3D maps. Scale bar: 1 μm.

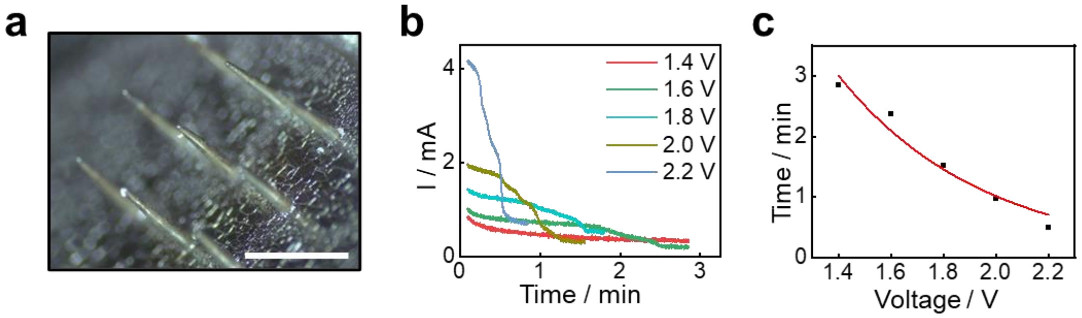

SI Figure 12. Characterization of electrochemical crevice corrosion of Mo-coated microneedles. a. Optical image of a 3 * 3 100-nm Mo-coated MN array (1.2-mm). Scale bar: 1 mm. **b.** Amperometry characterization of the electrochemical crevice corrosion of Mo layer on MNs under different potentials in 1X DPBS. **c.** The relationship between corrosion time and potential.

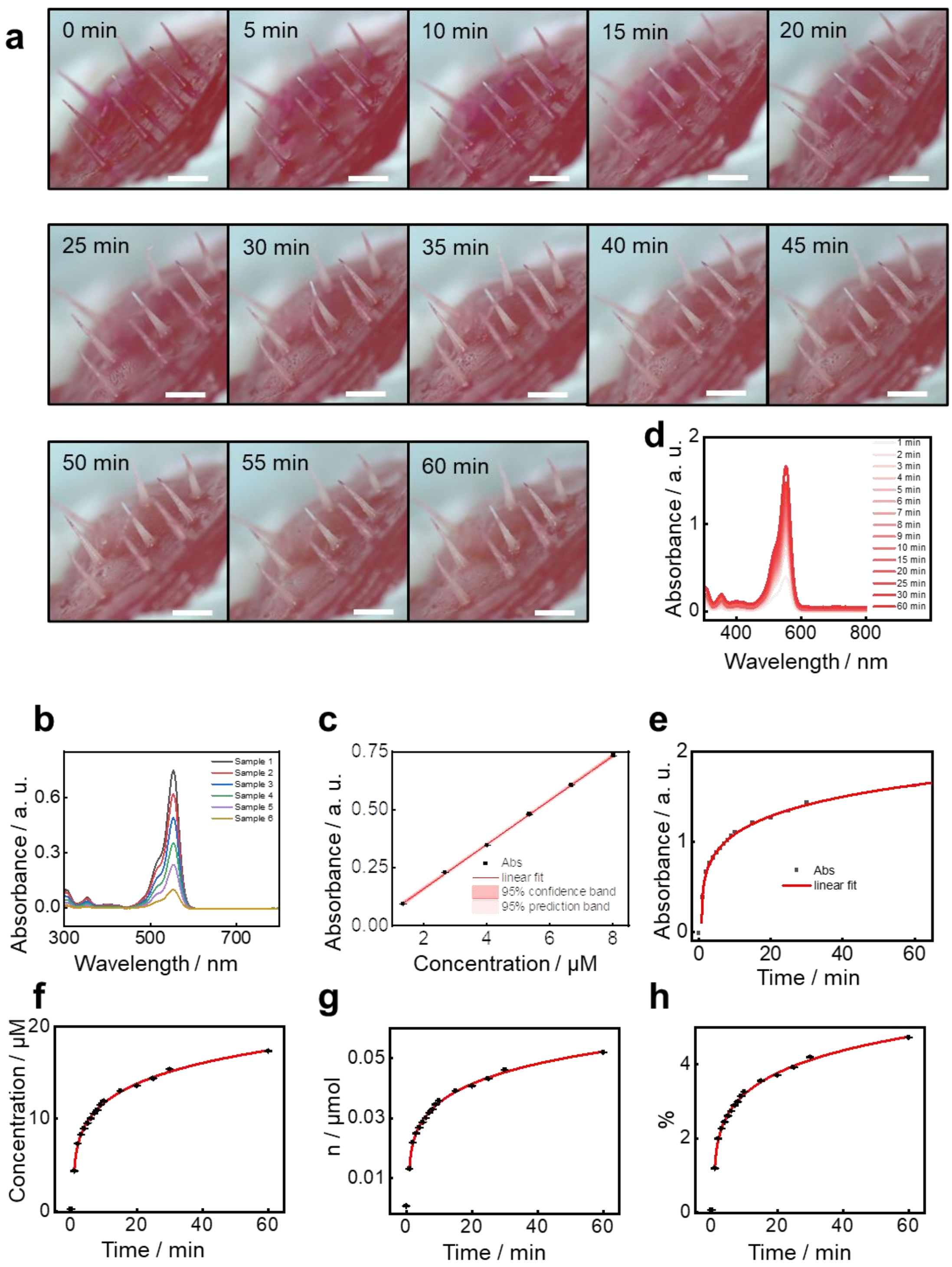

SI Figure 13. Characterization of dye release from microneedles (0.3% Rhodamine B loaded, 1.2-mm). a. Optical images of an MN array undergoing dye release from 0 to 60 minutes in 45 ℃ 1X DPBS. Scale bar: 500 μm. **b.** The UV-Vis spectroscopy (300-800 nm) of Rhodamine B standard solutions (Sample 1-6). **c.** The calibration curve of Rhodamine B from standard solutions in **b**. The calibration curve is calculated as: $\mathrm{Abs} = 0.09518 * \mathrm{C} - 0.02998$, where Abs is the absorption and C is the concentration. The standard error of the slope is $7.16908 * 10^{-4}$, and the

standard error of the intercept is 0.00373. The adjusted R^2 is 0.99972. The 95% confidence band and 95% prediction band are plotted together with the linear regression line. Data are presented as mean values +/- standard deviation of the peak absorption (Abs) based on the horizontal shifting analysis of peak. (n=6) **d.** Measured UV-Vis spectra of the environment solution corresponding to **a**. **e.** The absorbance versus time of environment solution from the dye release experiment. **f.-h.** The concentration, molar amount, and release ratio of Rhodamine B calculated based on calibration curve in **c** and the absorption data in **e**. Data are presented as predicted values +/- margin of error from 95% prediction interval. (n=6)

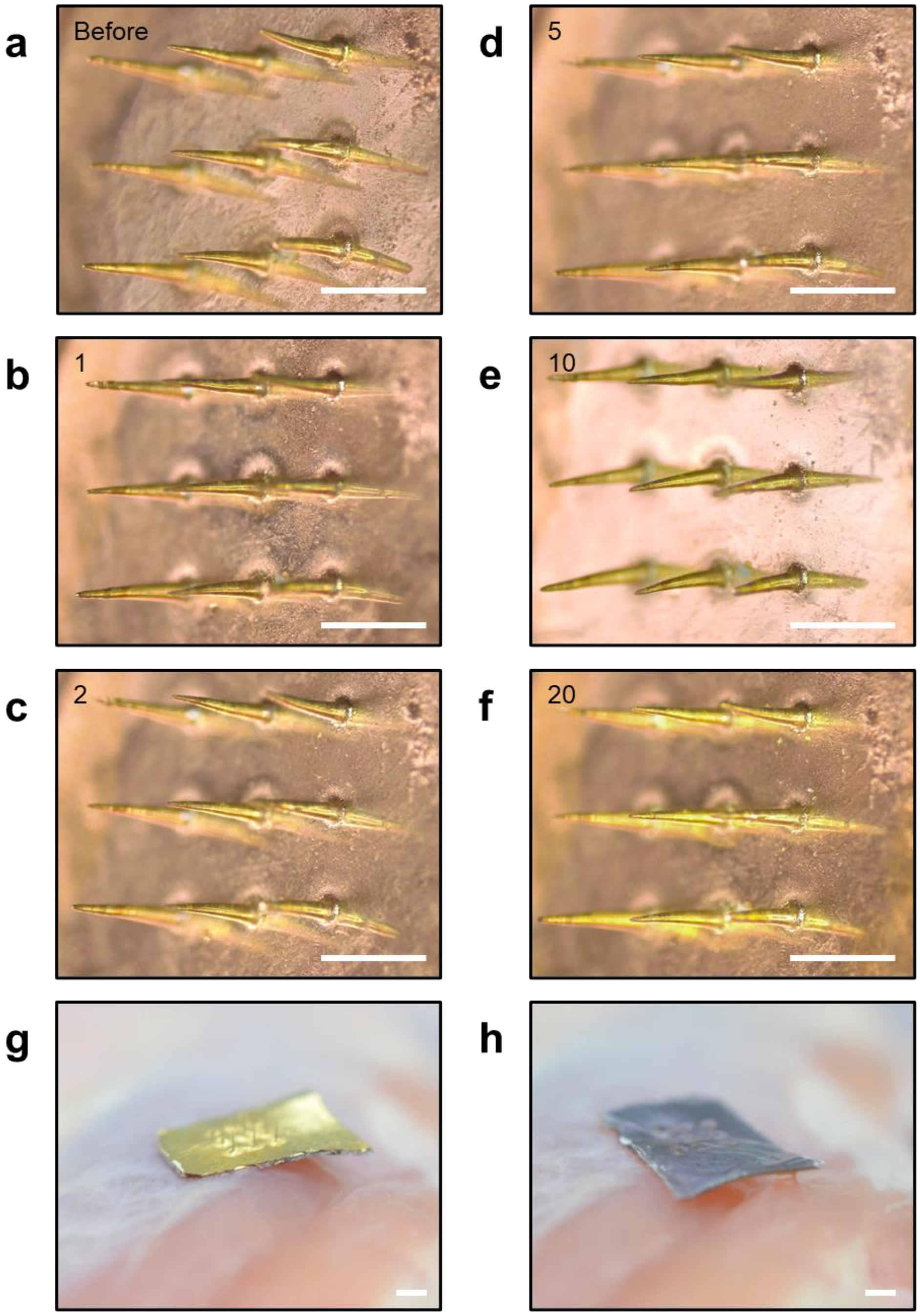

SI Figure 14. Optical images of gold-coated MN under penetration test. A 3*3 MN array (150 nm gold coated, 1.2 mm) is used in the penetration test of chicken thigh tissue. **a.** MN array before penetration. **b.** MN array after one penetration. **c.** MN array after 2 penetrations. **d.** MN array after 5 penetrations. **e.** MN array after 10 penetrations. **f.** MN array after 20 penetrations. **g.** The MN array facing upward on a chicken thigh. **h.** The MN array in contact with chicken thigh. Scale bars: 1 mm.

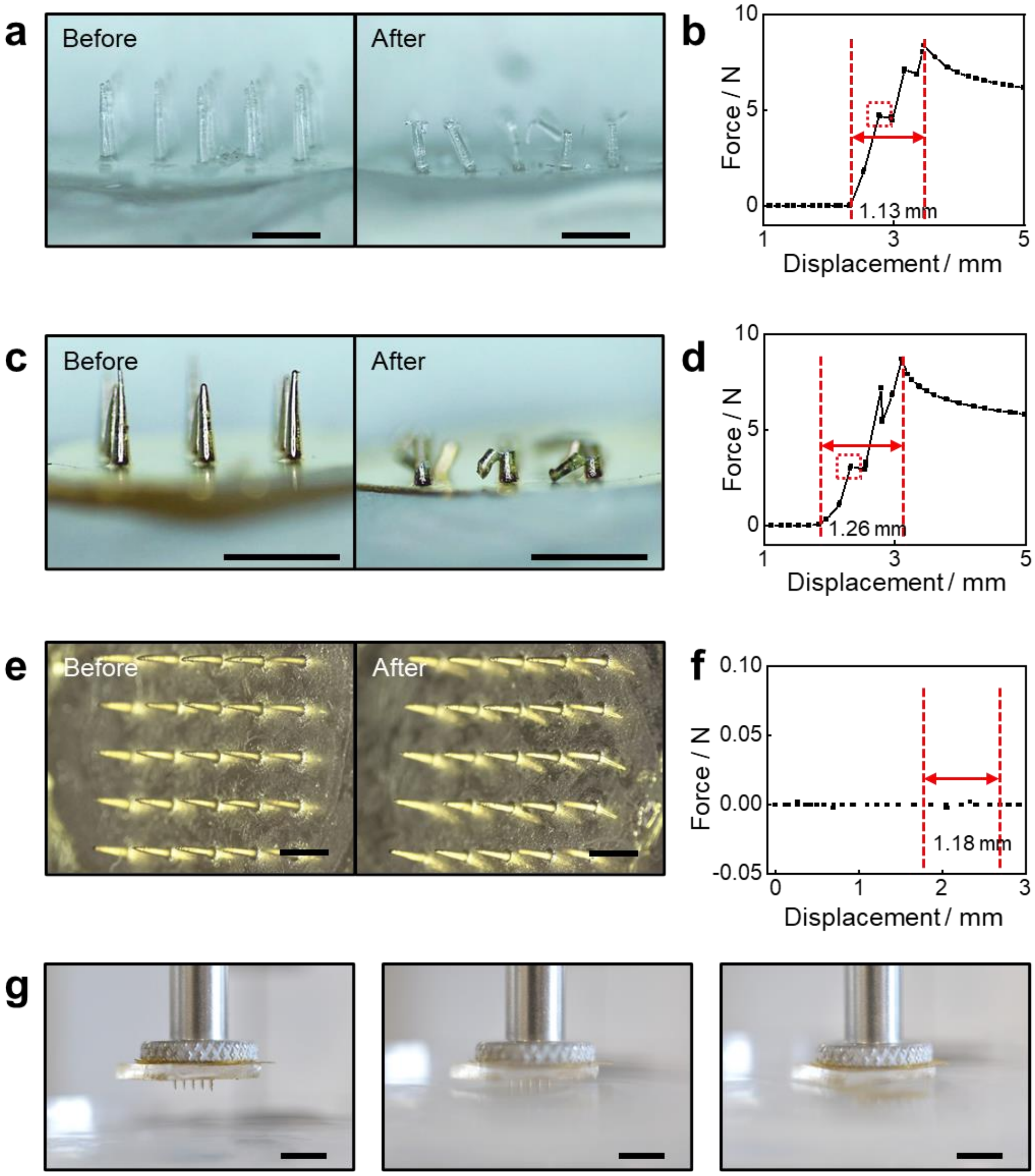

SI Figure 15. Mechanical characterization of microneedles. a. Optical images of a 5*5 1.5-mm MN array before and after fracture test. **b.** The mechanical testing curve for the 25-MN array, with the first mechanical failure circled in a red frame. The contact distance was labeled as 1.13 mm. **c.** Optical images of a 3*3 1.2-mm MN array before and after the fracture test. **d.** The mechanical testing curve for the 9-MN array, with the first mechanical failure circled in a red frame. The contact distance is labeled as 1.26 mm. **e.** Optical images of a 5*5 1.5-mm MN array before

and after the agarose penetration test. **f.** Mechanical testing curve for the 25-MN array on 0.5 % agarose. The contact distance is labeled as 1.18 mm. **g.** Optical images of the stages of agarose penetration test: before contact, partially penetrated, and fully penetrated. Scale bar: 1 mm in **a**, **c**, **e**, 5 mm in **g**. Similar results in SI Figure 15 are obtained from 3 experiments.

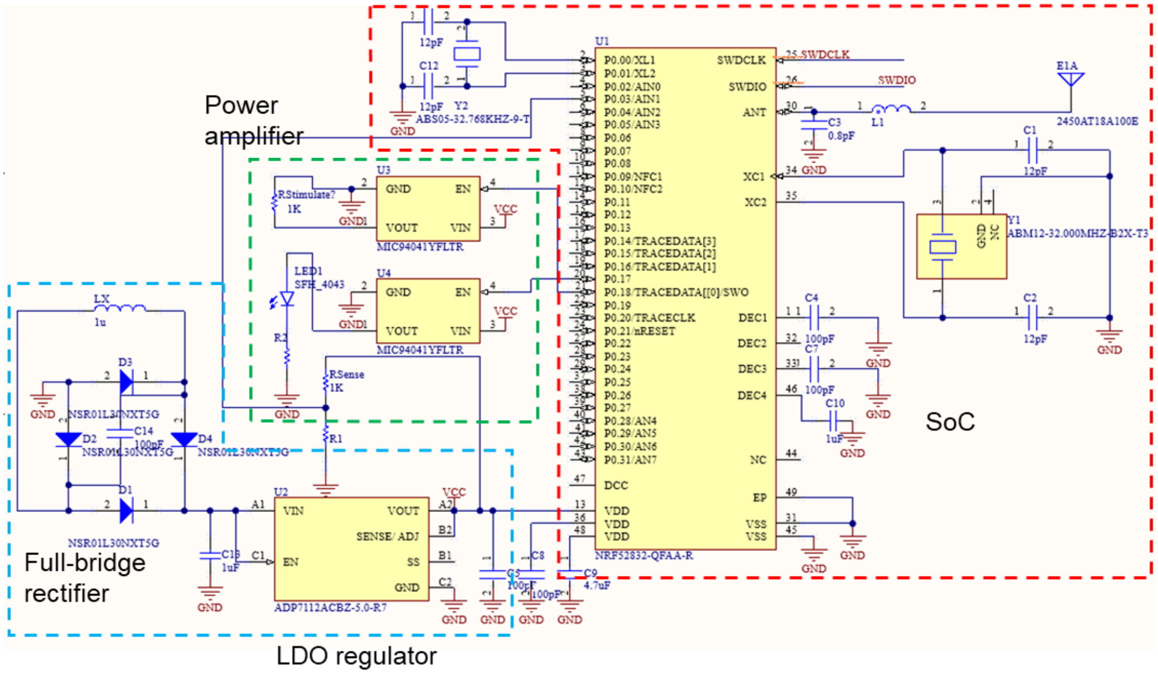

SI Figure 16. The circuit diagram of SOP with a wireless power harvesting system and a remote-control module. A power harvesting and signal conditioning circuit is fabricated and soldered using the method of soft PCB fabrications. A power harvesting network formed by a PCB inductor coil and a surface mount capacitor is used to harvest energy from the alternating magnetic flux generated from the inductively coupled transmission coil. The power harvesting network is connected to a full-bridge rectifier with surface-mount diodes, enabling the conversion of the harvested alternative current (AC) obtained via the receiver coil into direct current (DC). Subsequently, the rectified DC is fed into a 3.3 V low-dropout (LDO) regulator. The coil, rectifier, and regulator constitute the wireless energy harvesting system, which is circled in a blue frame. After the regulator, the output signal goes through a power amplification system, which is circled in green. The amplified output serves as the power source for a Bluetooth-Low-Energy (BLE) System-on-Chip (SoC). A custom BLE service programmed in the SoC enables clinicians to wirelessly and remotely control the general-purpose input/output (GPIO) modules through Bluetooth using computers or smartphones. This scheme, in conjunction with the programmed pulse width modulation (PWM) instance, onboard power amplifiers, and filtering circuits, facilitates programmable drug release by clinicians from the microneedle patch with high spatiotemporal resolution.

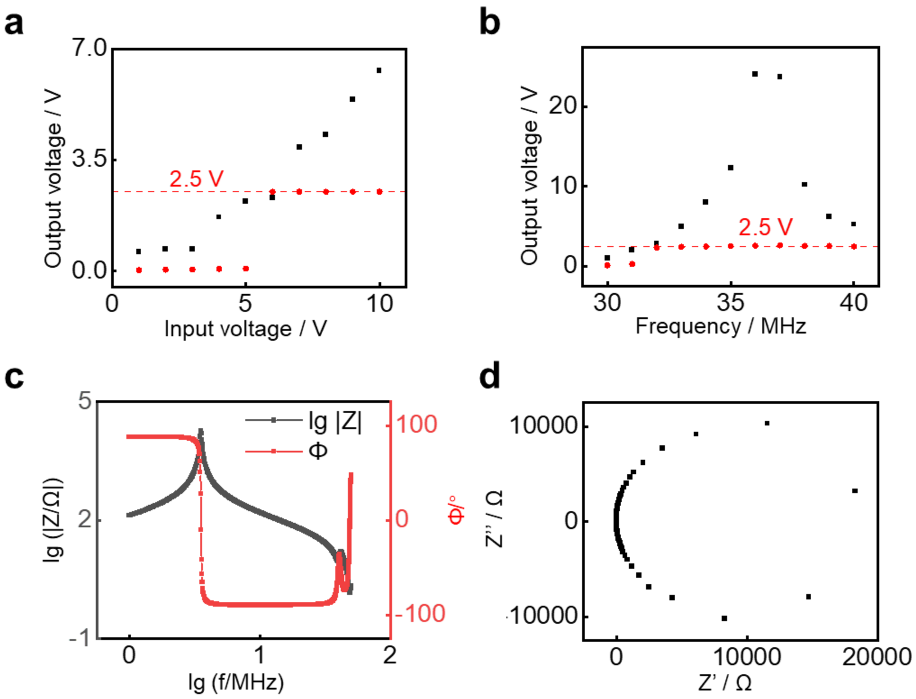

SI Figure 17. Characterization of the wireless power transfer module for SOP. a. Power transfer efficiency at 40 MHz. The input signal refers to the signal applied on the inductive coil, while the output signal refers to the signal after full-bridge rectification (black) and 2.5-V regulation (red). A minimum input peak-to-peak voltage of 7 V is required to generate stable 2.5 V DC potential. **b.** Output voltage versus frequency with 10-V (peak-to-peak) input signal, indicating an optimal transmitting frequency at around 36 MHz. Black, rectified output signal; red, 2.5-V regulated output signal. **c. d.** Bode plot and Nyquist plot of the SOP system.

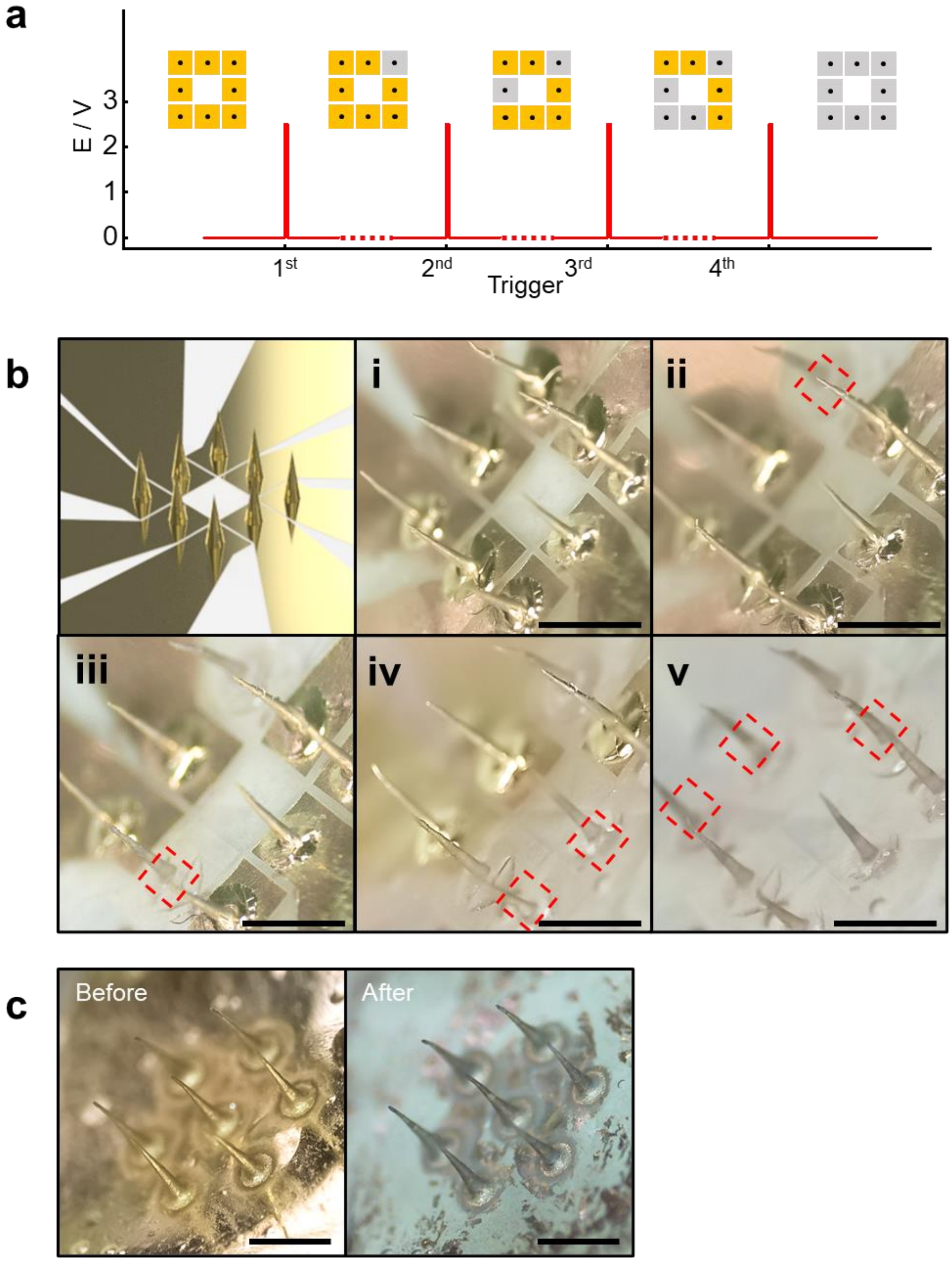

SI Figure 18. Characterization of stepwise release control on single microneedles. a. Schematic illustration of the electrical triggers at the single-needle level. The MNs (1.2-mm, 150 nm gold coated) on the array are separated from each other and triggered, respectively. **b.** The 8-needle device demonstration and optical images of the multistage triggering (Stage **i** to Stage **v**) on single MNs of the patch. The MNs triggered at the previous stage are labeled by red frames. **c.** Optical images of a 7-needle array before and after electrical triggering. Scale bar: 1 mm.

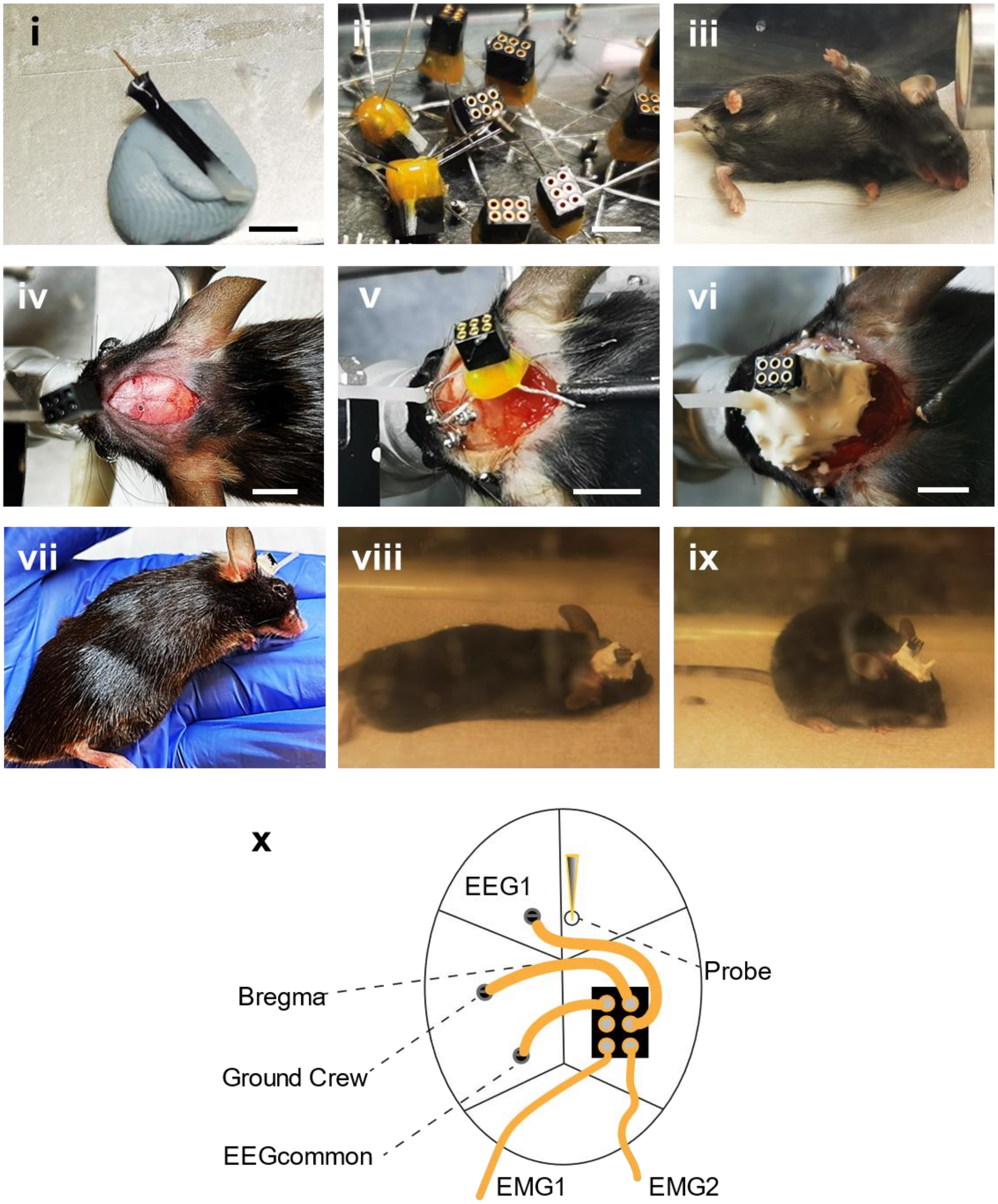

SI Figure 19. Implantation surgery of in vivo microneedles and electrodes. i. Mounting the MN on a 3D-printed rod. **ii.** Preparation of electrodes for EEG/EMG recording. Header pitch is 1 mm. **iii.** Anesthesia of the mouse by isoflurane. **iv.** Fixing the head of the mouse on a clamp platform. The scalp is partially exposed to insert EEG/EMG electrodes and a hole is drilled for the microneedle. **v.** Implantation of the microneedle and electrodes. **vi.** Encapsulation of microneedles and electrodes. **vii-viii.** Post-surgery anesthesia recovery. **ix.** Recovered mouse bearing with the microneedle and electrodes. **x.** The electrode configuration map. The positions

of two EMG electrodes (EMG1, EMG2), an EEG electrode (EEG1), a ground electrode (Ground Crew), the EEG common electrode, and the microneedle (Probe) are labeled. Scale bar: 3 mm.

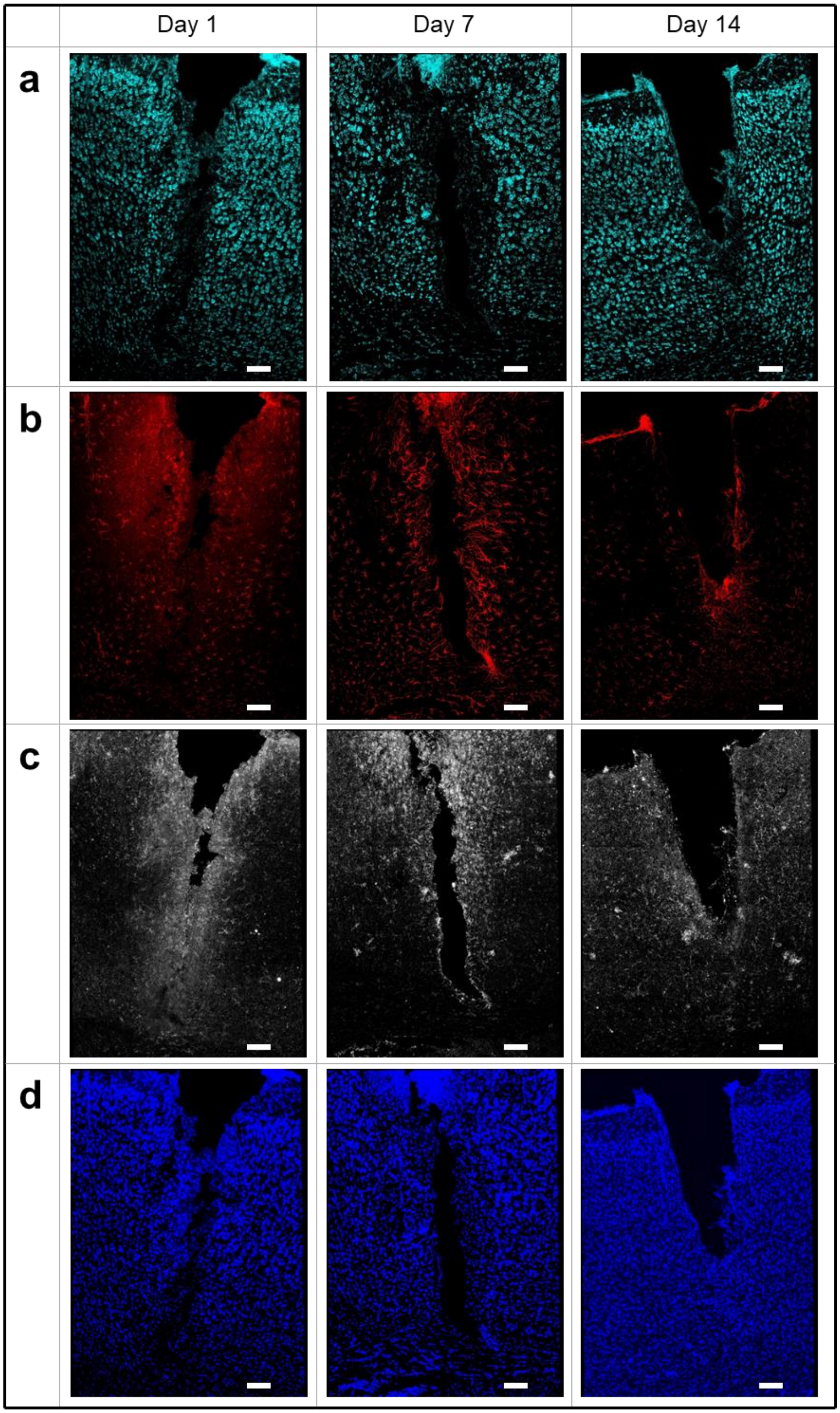

SI Figure 20. Representative confocal images of 40-μm horizontal cortical slices at various stages after implantation of the SOP microneedles. Probes were collected on days 1, 7, and 14, covering the typical lifetime of a SOP microneedle. The images show cross-sectional views of the implantation site with immunohistochemical staining for: **a.** Nissl bodies (neurotrace, green); **b.** astrocytes (glial fibrillary acidic protein (GFAP), red); **c.** activated microglia (Iba1, white); and **d.** DNA (4′,6-diamidino-2-phenylindole (DAPI), blue), and overall lesions from bioresorbable optical probes (n = 3 independent experiments). Scale bar: 100 μm.

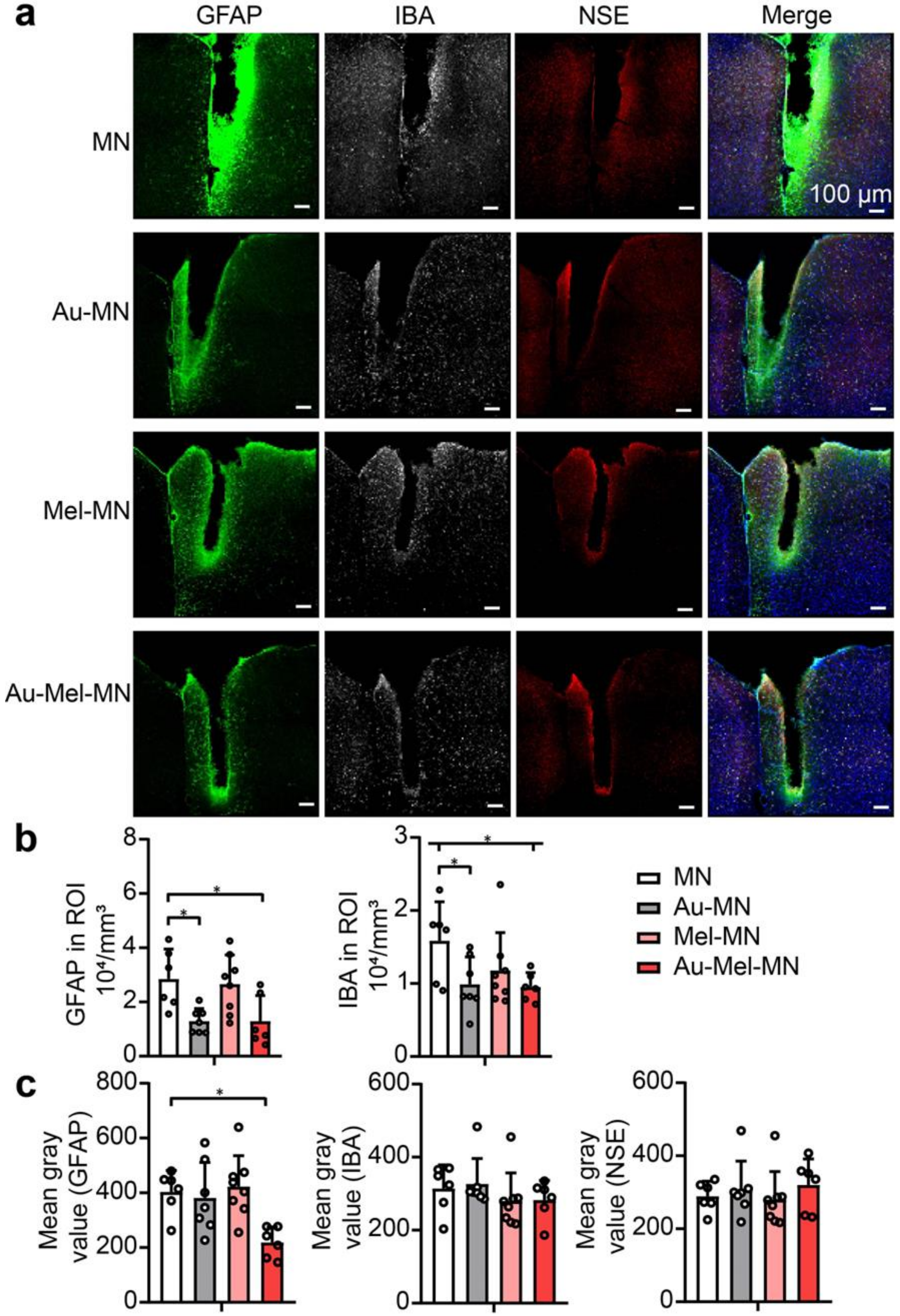

a
GFAP
IBA
NSE
Merge
MN
Au-MN
Mel-MN
Au-Mel-MN
100 μm
b
GFAP in ROI 10⁴/mm³
IBA in ROI 10⁴/mm³
MN
Au-MN
Mel-MN
Au-Mel-MN
c
Mean gray value (GFAP)
Mean gray value (IBA)
Mean gray value (NSE)

SI Figure 21. Immunohistochemical analysis to validate biocompatibility of SOP. The study compared four groups of MNs, including bare MN (MN), gold-coated MN (Au-MN), melatonin-loaded MN (Mel-MN), and melatonin-loaded MN with gold-coating (Au-Mel-MN). **a.** Representative confocal images of 40-μm horizontal cortical slices after intracranial implantation of the MNs in mice. The images show cross-sectional views of the implantation site with immunohistochemical staining for 1) astrocytes (glial fibrillary acidic protein (GFAP), green); 2) activated microglia (Iba1, white); 3) Neuron-specific enolase (NSE, red) and 4) DNA (4′,6-diamidino-2-phenylindole (DAPI), blue), and overall lesions from bioresorbable MNs. **b.** Measured the number of astrocytes and microglia in the region of interest (ROI). **c.** Average fluorescence intensity of astrocytes and microglia in the region of interest (ROI). (n = 6-8 in each group). $*p < 0.05$. One-way ANOVA followed by PLSD post hoc test. Scale bar: 100 μm.

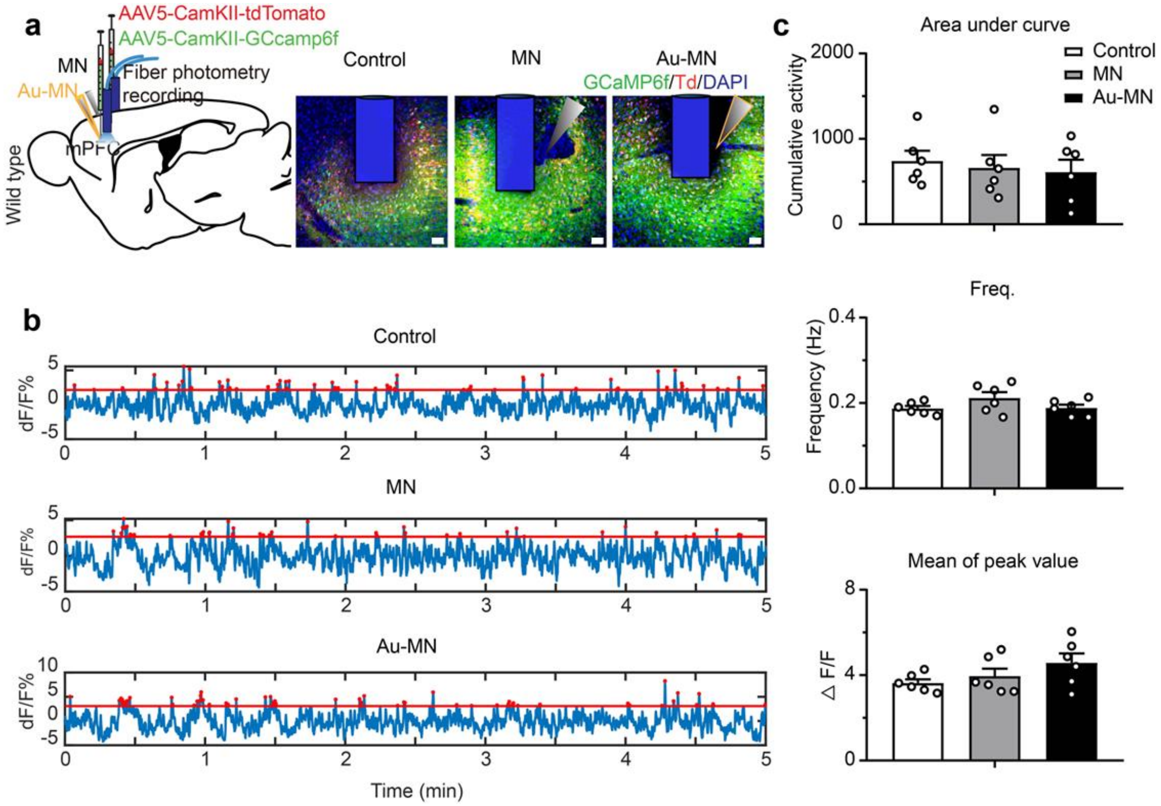

SI Figure 22. Recorded Ca^{2+} activities of the prefrontal cortex (PFC) after sham operation and implantation of the bare or the gold-coated MNs. a. The schematic illustration and fluorescence images that shows fiber photometry recording after intracranial implantation of MNs in mice. Scale bar: 50 μm. The positions of the optical fiber and MN are labeled. GCaMP6f, Td, and DAPI are in green, red, and blue. **b.** Typical GCaMP6f traces (5 mins) of the sham group (Control, upper), the group with bare MNs (MN, middle), and the group with the gold-coated MNs (Au-MN, bottom) from the PFC. **c.** The cumulative activity, frequency, and average peak value of DF/F of the PFC calcium signals. (n = 6 in each group)

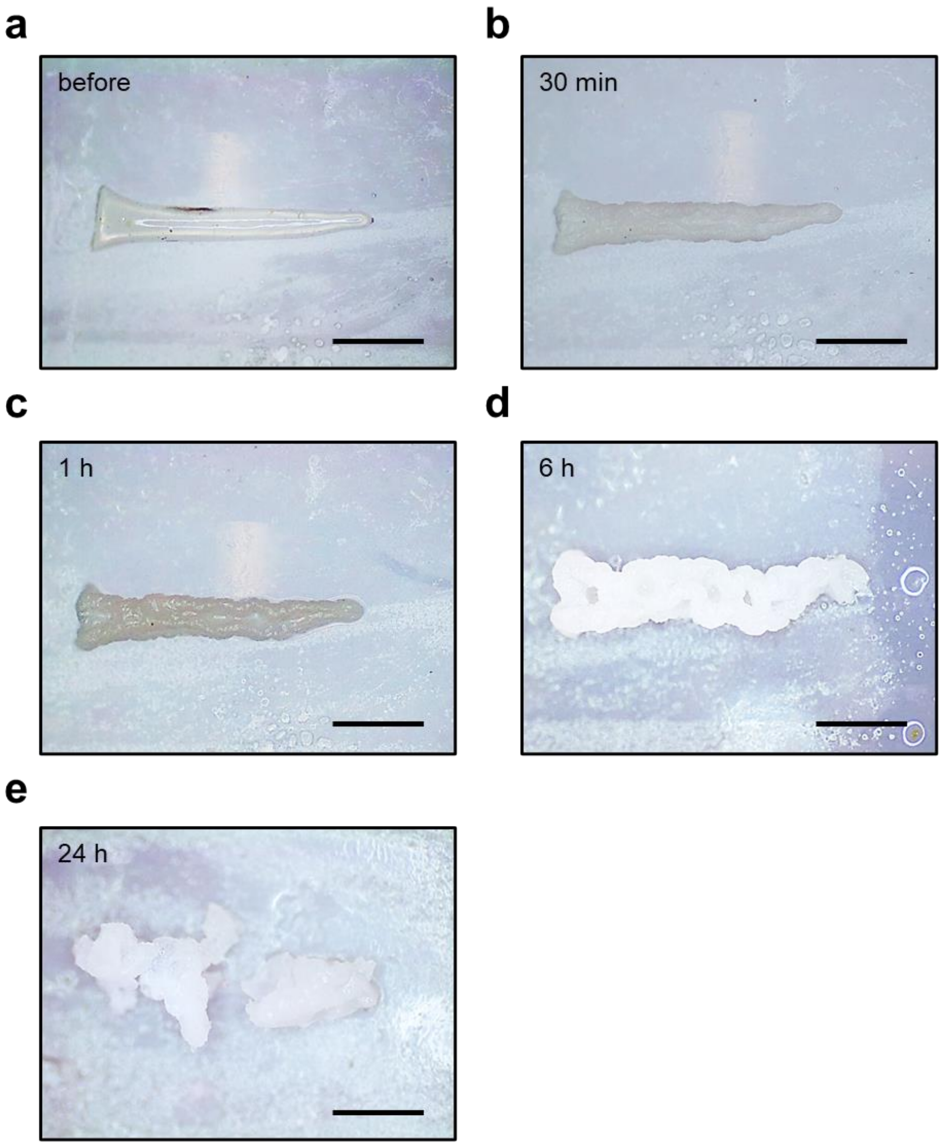

SI Figure 23. PLGA MN degradation in PBS. The PLGA MN (3 mm) is soaked in PBS at 65 ℃ as an accelerated degradation experiment. Optical images by microscope are taken before degradation (a), 30 minutes after degradation (b), 1 hour after degradation (c), 6 hours after degradation (d), and 24 hours after degradation. Scale bar: 1 mm. Similar results are obtained from 3 experiments.

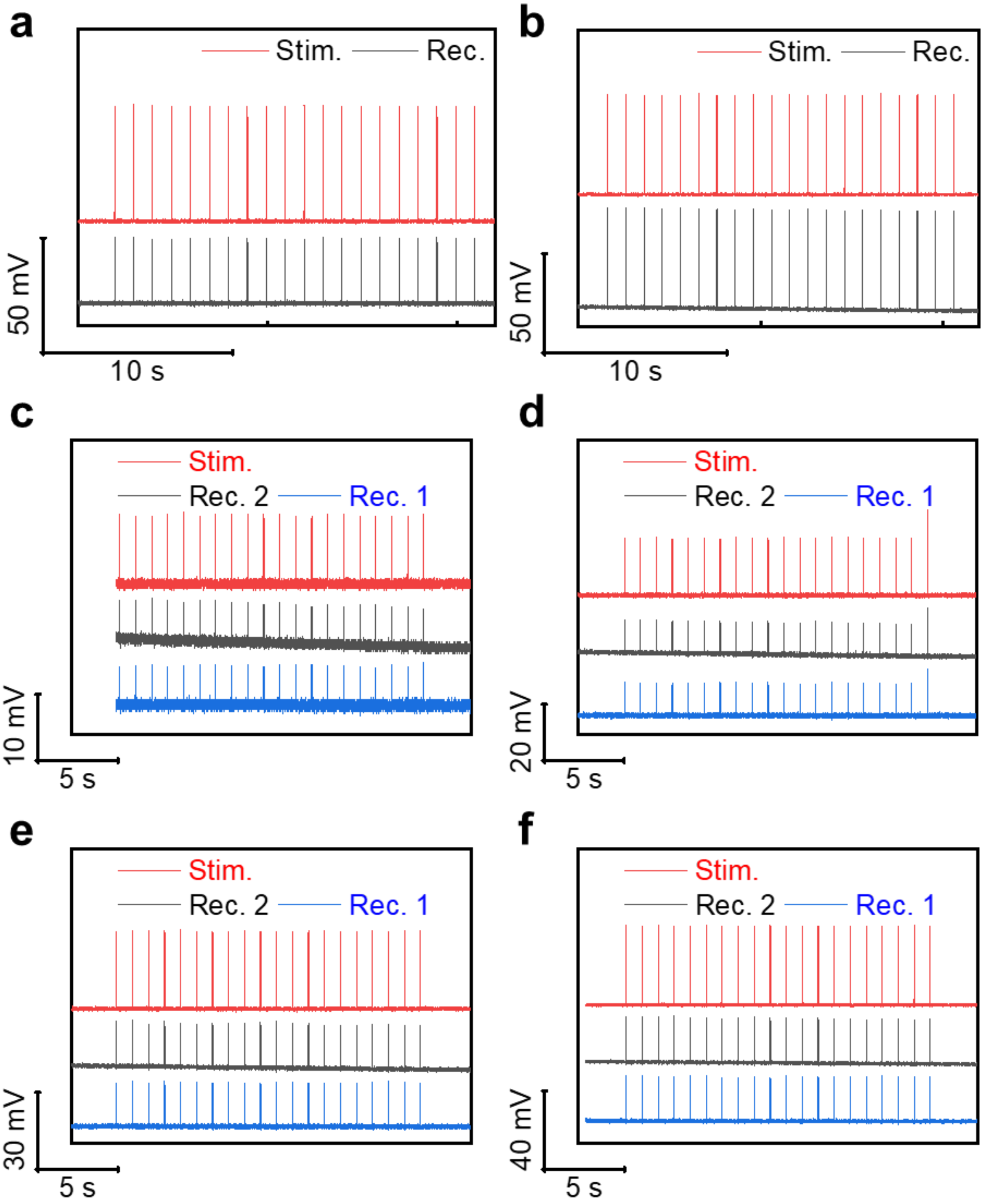

a
Stim.
Rec.
50 mV
10 s
b
Stim.
Rec.
50 mV
10 s
c
Stim.
Rec. 2
Rec. 1
10 mV
5 s
d
Stim.
Rec. 2
Rec. 1
20 mV
5 s
e
Stim.
Rec. 2
Rec. 1
30 mV
5 s
f
Stim.
Rec. 2
Rec. 1
40 mV
5 s

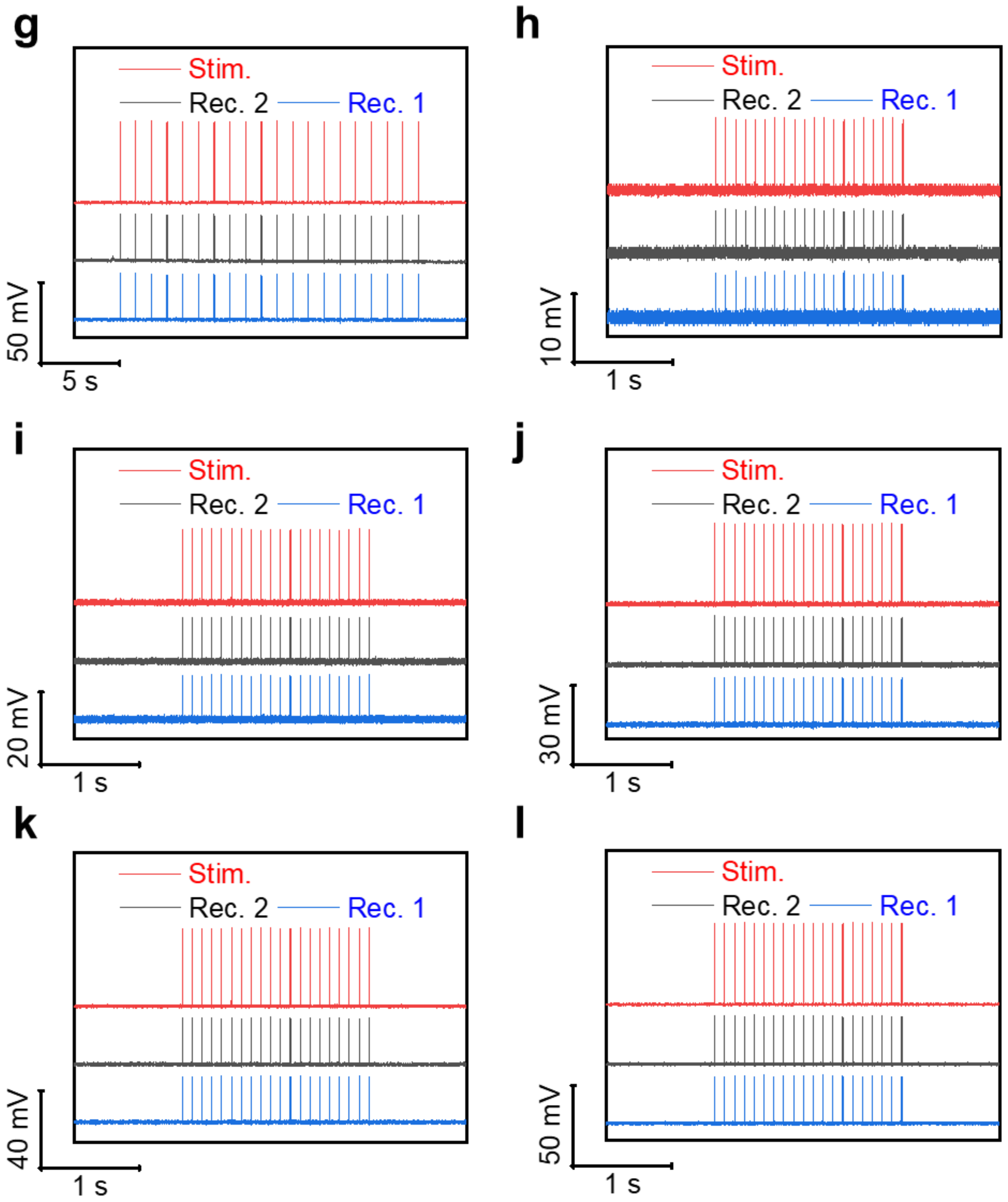

SI Figure 24. Brain stimulation with microneedles (3-mm, 150-nm gold coated). a. b. The signal (50 mV, 1 Hz, 10 ms in duration) recorded (Rec.) 5 mm (**a**) and 2 mm (**b**) away from the stimulation MN (Stim.) **c.-g.** The 1 Hz stimulation (10 ms in duration) from 10-50 mV recorded by two electrodes (Rec. 1 and Rec. 2, ~5 mm away from the MN). **h.-l.** The 10 Hz stimulation (1 ms in duration) from 10-50 mV recorded by two electrodes (Rec. 1 and Rec. 2, ~5 mm away from the MN).

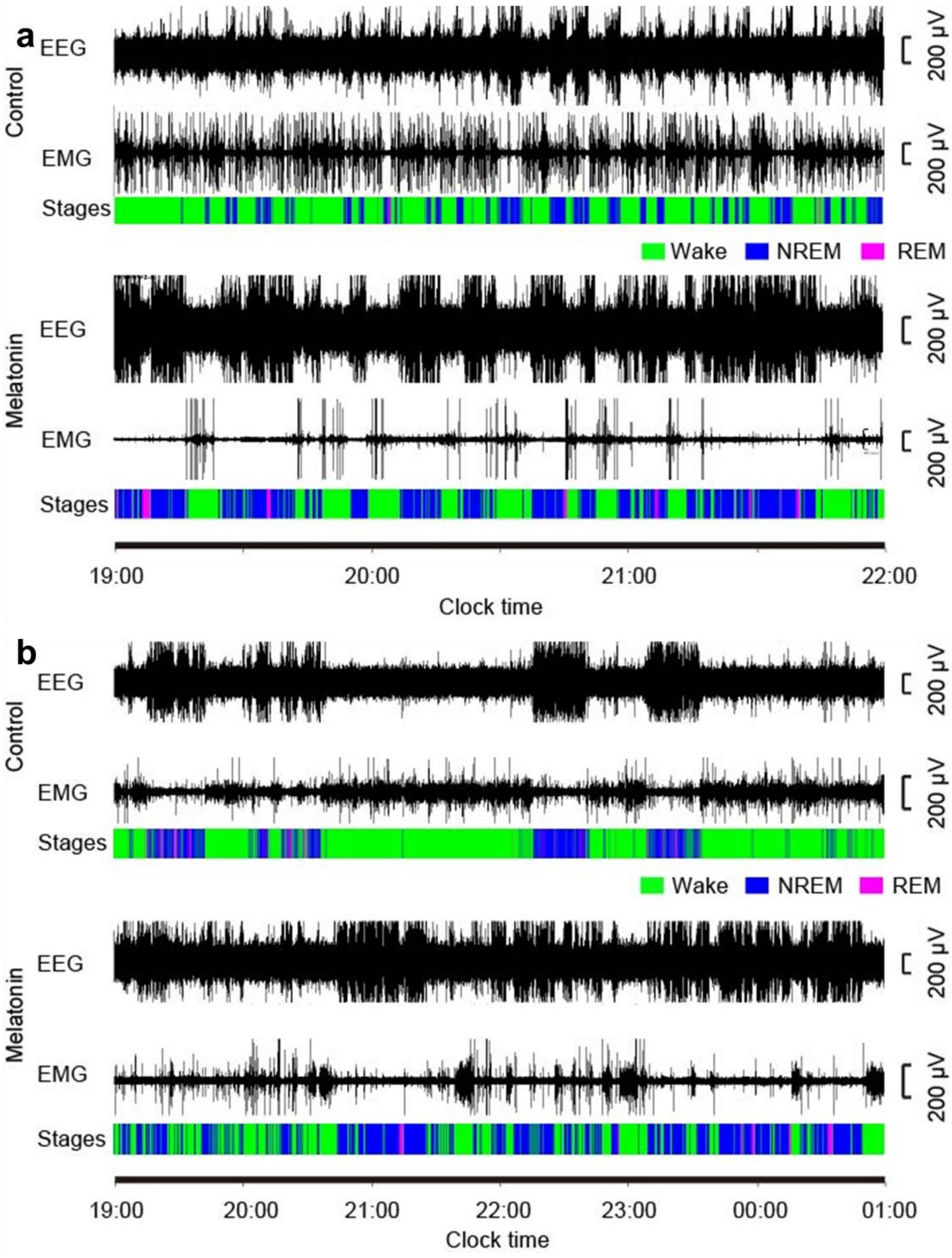

SI Figure 25. In vivo recording of EEG and EMG. a. The recording shows the EEG/EMG traces and hypnograms from both the Mel-MN and the control group, during the 3-hour period from 19:00-22:00. Green: wake status; blue: non-rapid eye movement status; purple: rapid eye movement status. **b.** The recording shows the EEG/EMG traces and hypnograms from both the Au-Mel-MN and the control group, during the 6-hour period from 19:00-01:00. Green: wake status;

blue: non-rapid eye movement status; purple: rapid eye movement status. It is shown that NREM length is higher in melatonin-releasing groups (Mel-MN and Au-Mel-MN) than that in the control groups.

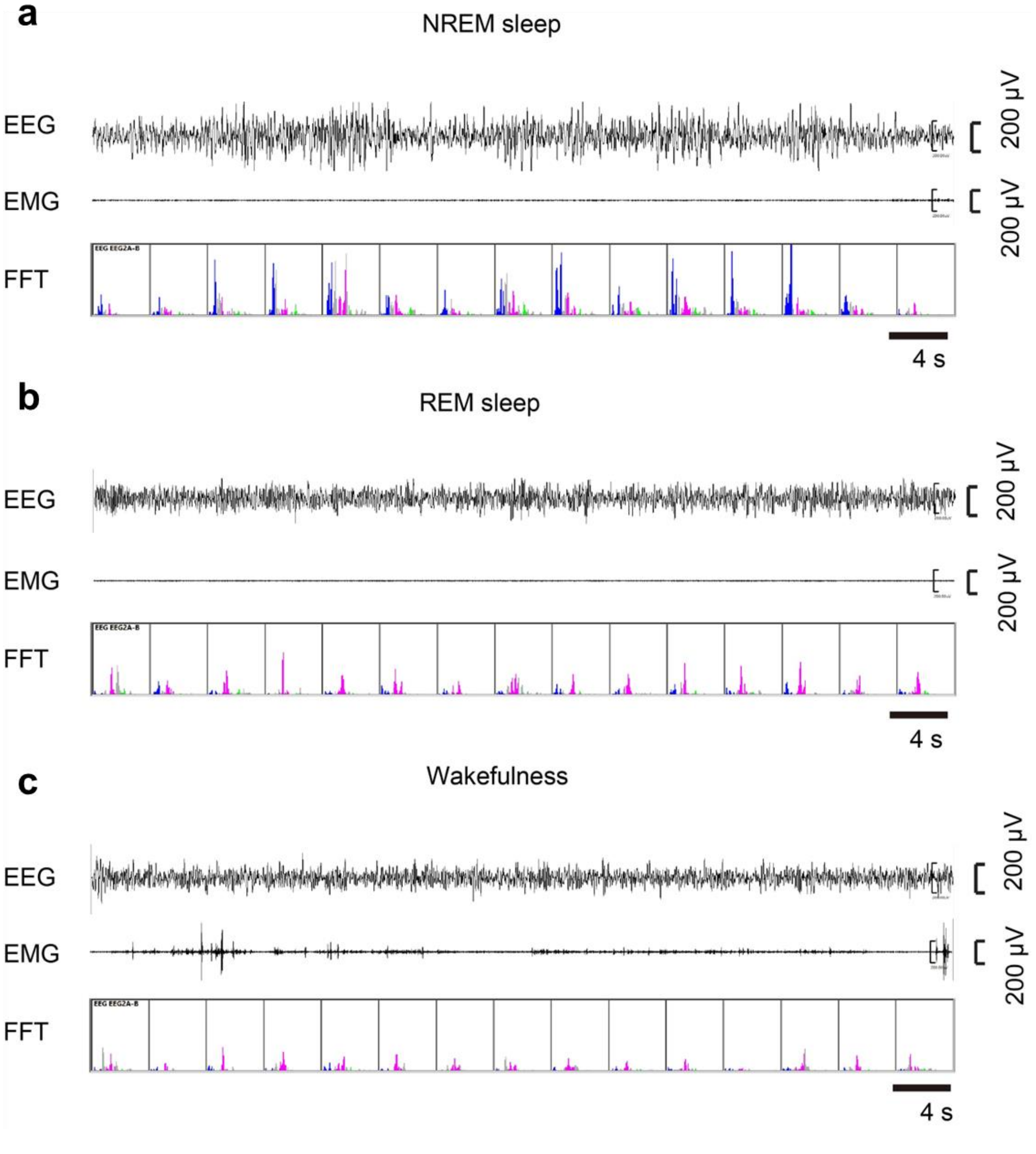

SI Figure 26. The typical signal patterns of three statuses (NREM, REM, Wake) of the Mel-MN control group (without melatonin payloads). a. The EEG, EMG, and Fast-Fourier Transform (FFT) pattern of a 1-min slice of NREM sleep period. The FFT is calculated every 4 s in the 1-min slice. **b.** The EEG, EMG, and FFT pattern of a 1-min slice of REM sleep period. **c.** The EEG, EMG, and FFT pattern of 1-min slice of wakefulness period. In the FFT pattern, the delta wave (0.1-4 Hz) is highlighted in blue, the theta wave (6-9 Hz) is highlighted in pink, and the alpha (8-12 Hz) wave is highlighted in green.

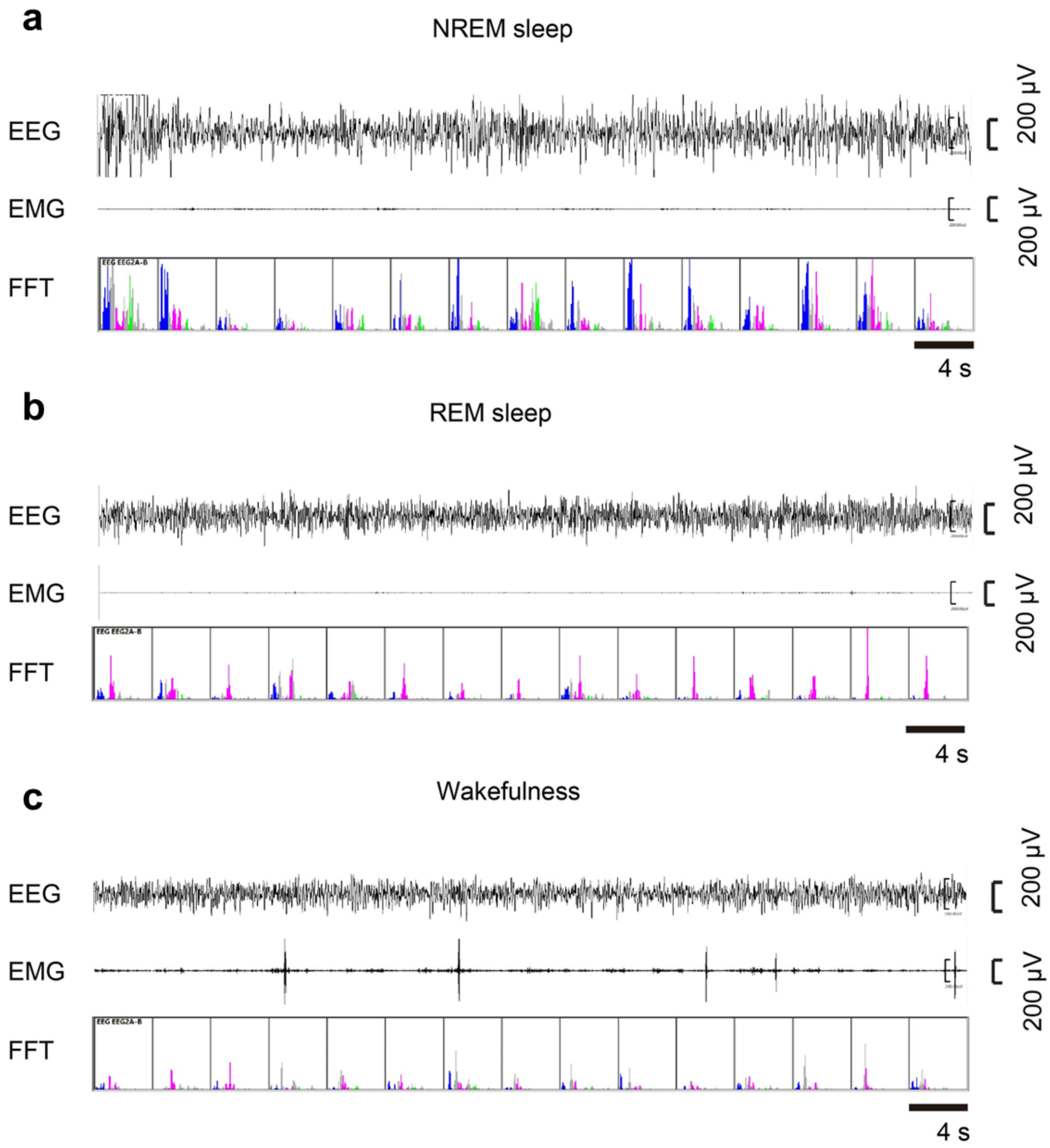

SI Figure 27. The typical signal patterns of three statuses (NREM, REM, Wake) of the Mel-MN releasing group (with melatonin payloads). a. The EEG, EMG, and Fast-Fourier Transform (FFT) pattern of a 1-min slice of NREM sleep period. The FFT is calculated every 4 s in the 1-min slice. **b.** The EEG, EMG, and FFT pattern of a 1-min slice of REM sleep period. **c.** The EEG, EMG, and FFT pattern of 1-min slice of wakefulness period. In the FFT pattern, the delta wave (0.1-4 Hz) is highlighted in blue, the theta wave (6-9 Hz) is highlighted in pink, and the alpha (8-12 Hz) wave is highlighted in green.

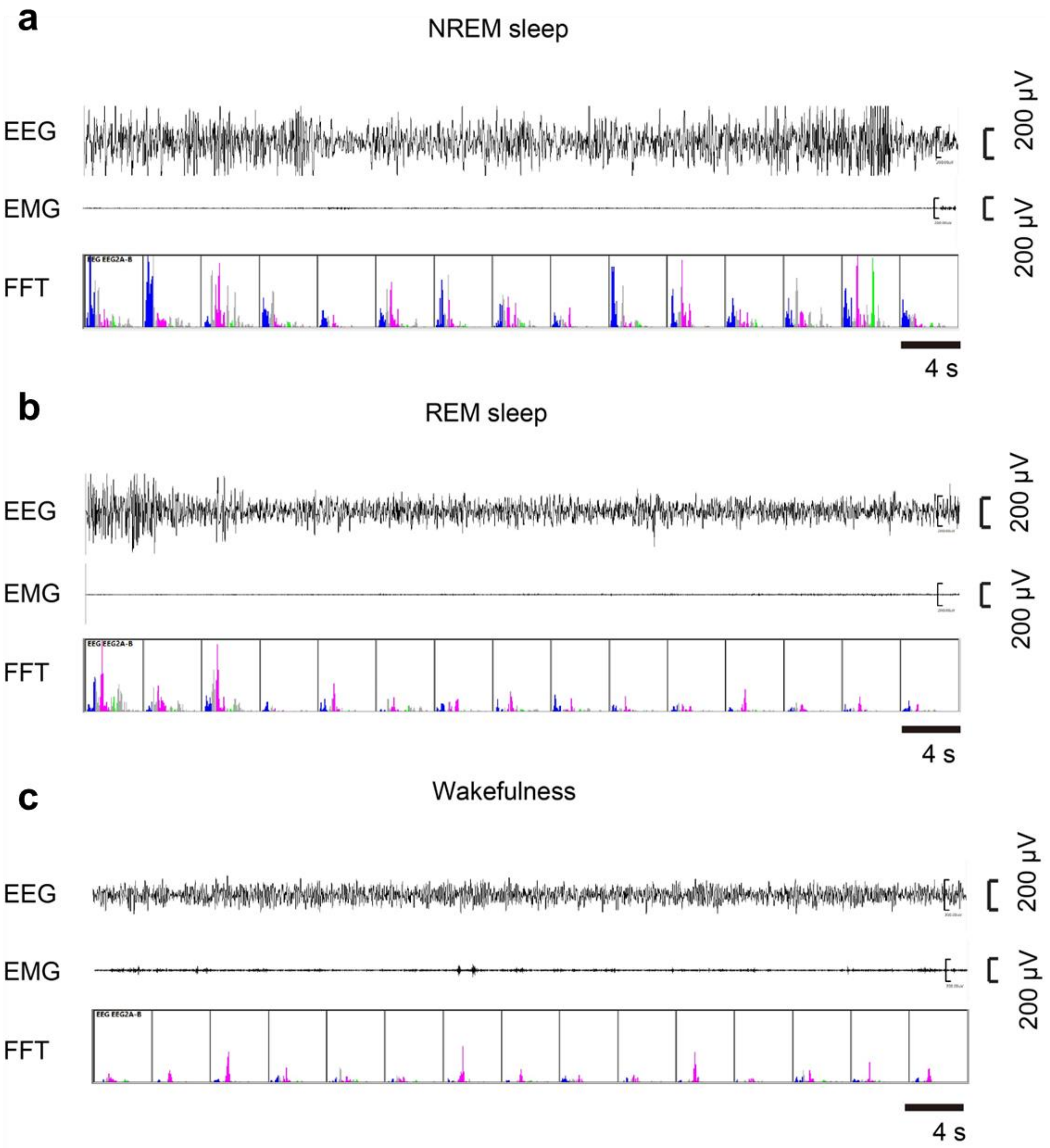

SI Figure 28. The typical signal patterns of three statuses (NREM, REM, Wake) of the Au-Mel-MN control group (without melatonin payloads). a. The EEG, EMG, and Fast-Fourier Transform (FFT) pattern of a 1-min slice of NREM sleep period. The FFT is calculated every 4 s in the 1-min slice. **b.** The EEG, EMG, and FFT pattern of a 1-min slice of REM sleep period. **c.** The EEG, EMG, and FFT pattern of 1-min slice of wakefulness period. In the FFT pattern, the delta wave (0.1-4 Hz) is highlighted in blue, the theta wave (6-9 Hz) is highlighted in pink, and the alpha (8-12 Hz) wave is highlighted in green.

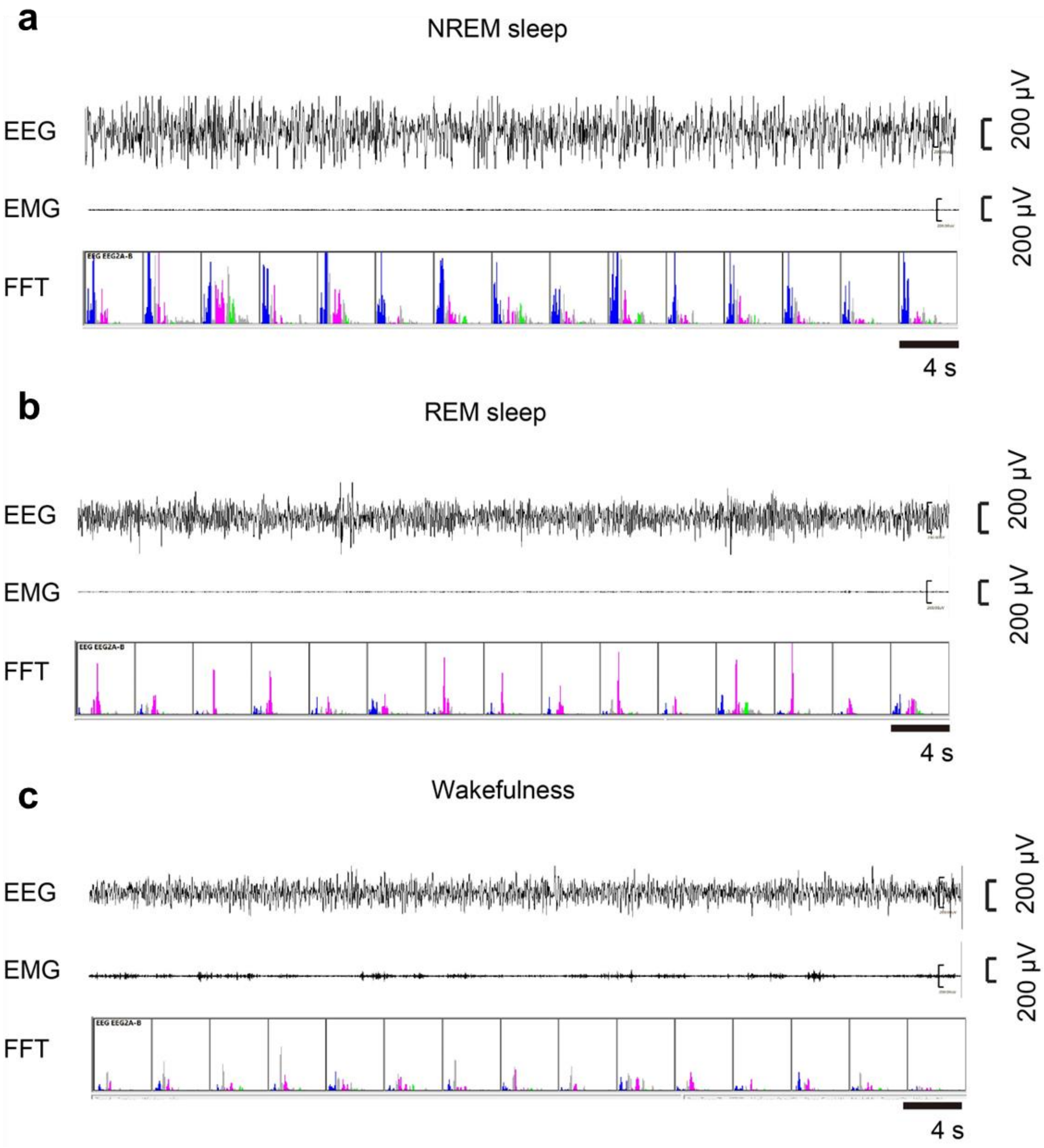

SI Figure 29. The typical signal patterns of three statuses (NREM, REM, Wake) of the Au-Mel-MN releasing group (with melatonin payloads). a. The EEG, EMG, and Fast-Fourier Transform (FFT) pattern of a 1-min slice of NREM sleep period. The FFT is calculated every 4 s in the 1-min slice. **b.** The EEG, EMG, and FFT pattern of a 1-min slice of REM sleep period. **c.** The EEG, EMG, and FFT pattern of 1-min slice of wakefulness period. In the FFT pattern, the delta wave (0.1-4 Hz) is highlighted in blue, the theta wave (6-9 Hz) is highlighted in pink, and the alpha (8-12 Hz) wave is highlighted in green.

Time / min	n(RhB) / µmol – W/O Au	n(RhB) / µmol – W/ Au
0	0.000610843	0.000610843
1	0.013033831	0.00059067
2	0.021904602	0.000601072
3	0.024912166	0.000608636
4	0.026847132	0.000607691
5	0.028640891	0.000619353
6	0.03008258	0.00067262
7	0.031771065	0.000660328
8	0.032870141	0.000650557
9	0.034568397	0.000657176
10	0.035853436	0.000967703
15	0.039121034	0.000699412
20	0.040754991	0.000747321
25	0.043185438	0.000677979
30	0.046155495	0.000704455
60	0.052038874	0.001282517

SI Table 1. The accumulative release amount of Rhodamine B (RhB). This table corresponds to the RhB release experiment described in Figure 3b and SI Figure 13. Both groups, microneedle array without and with gold encapsulation (W/O Au and W/ Au) are calculated for every recorded time point during the 60-min release.
