# Supplementary material for: Digital automation of transdermal drug delivery with high spatiotemporal resolution
Source: Nat Commun. 2024 Jan 13;15:511. doi: 10.1038/s41467-023-44532-0 (PMC10787768; doi:10.1038/s41467-023-44532-0)
Supplement: Supplementary file 3 — Description of Additional Supplementary Files [file 41467_2023_44532_MOESM3_ESM.pdf]

<u>Description of Additional Supplementary Files</u>

Supplementary Movie 1. The real-time video demonstration of the stepwise electrical triggering on the MN patch of multiple domains. This video corresponds to the experiment described in Figure 4
