# Supplementary material for: Digital automation of transdermal drug delivery with high spatiotemporal resolution
Source: Nat Commun. 2024 Jan 13;15:511. doi: 10.1038/s41467-023-44532-0 (PMC10787768; doi:10.1038/s41467-023-44532-0)
Supplement: Supplementary file 5 — Reporting Summary [file 41467_2023_44532_MOESM5_ESM.pdf]

nature portfolio

Corresponding author(s): Juan Song, Wubin Bai

Last updated by author(s): Nov 26, 2023

Reporting Summary

Nature Portfolio wishes to improve the reproducibility of the work that we publish. This form provides structure for consistency and transparency in reporting. For further information on Nature Portfolio policies, see our Editorial Policies and the Editorial Policy Checklist.

Statistics

For all statistical analyses, confirm that the following items are present in the figure legend, table legend, main text, or Methods section.

n/a	Confirmed	
☐	☒	The exact sample size (*n*) for each experimental group/condition, given as a discrete number and unit of measurement
☐	☒	A statement on whether measurements were taken from distinct samples or whether the same sample was measured repeatedly
☐	☒	The statistical test(s) used AND whether they are one- or two-sided<br>*Only common tests should be described solely by name; describe more complex techniques in the Methods section.*
☐	☒	A description of all covariates tested
☐	☒	A description of any assumptions or corrections, such as tests of normality and adjustment for multiple comparisons
☐	☒	A full description of the statistical parameters including central tendency (e.g. means) or other basic estimates (e.g. regression coefficient) AND variation (e.g. standard deviation) or associated estimates of uncertainty (e.g. confidence intervals)
☐	☒	For null hypothesis testing, the test statistic (e.g. *F*, *t*, *r*) with confidence intervals, effect sizes, degrees of freedom and *P* value noted<br>*Give P values as exact values whenever suitable.*
☒	☐	For Bayesian analysis, information on the choice of priors and Markov chain Monte Carlo settings
☒	☐	For hierarchical and complex designs, identification of the appropriate level for tests and full reporting of outcomes
☒	☐	Estimates of effect sizes (e.g. Cohen's *d*, Pearson's *r*), indicating how they were calculated

Our web collection on statistics for biologists contains articles on many of the points above.

Software and code

Policy information about availability of computer code

Data collection	LabChart 8, NI LabView 2022, VWR UV software, FV31s-VW (Olympus FV3000), Sirenia Acquisition (Pinnacle)
Data analysis	Origin Pro 2022, ImageJ (Fiji, Java 1.8.0_172), GraphPad Prism 8, Matlab R2022b, SleepSign (Kissei Comtec, Nagano, Japan)

For manuscripts utilizing custom algorithms or software that are central to the research but not yet described in published literature, software must be made available to editors and reviewers. We strongly encourage code deposition in a community repository (e.g. GitHub). See the Nature Portfolio guidelines for submitting code & software for further information.

Data

Policy information about availability of data

All manuscripts must include a data availability statement. This statement should provide the following information, where applicable:

- Accession codes, unique identifiers, or web links for publicly available datasets
- A description of any restrictions on data availability
- For clinical datasets or third party data, please ensure that the statement adheres to our policy

The data of this study are available within the article, the Supplementary Information. Source data are provided with this paper and available upon request.

Research involving human participants, their data, or biological material

Policy information about studies with human participants or human data. See also policy information about sex, gender (identity/presentation), and sexual orientation and race, ethnicity and racism.

Reporting on sex and gender	N/A
Reporting on race, ethnicity, or other socially relevant groupings	N/A
Population characteristics	N/A
Recruitment	N/A
Ethics oversight	N/A

Note that full information on the approval of the study protocol must also be provided in the manuscript.

Field-specific reporting

Please select the one below that is the best fit for your research. If you are not sure, read the appropriate sections before making your selection.

☒ Life sciences ☐ Behavioural & social sciences ☐ Ecological, evolutionary & environmental sciences

For a reference copy of the document with all sections, see nature.com/documents/nr-reporting-summary-flat.pdf

Life sciences study design

All studies must disclose on these points even when the disclosure is negative.

Sample size	The sample size used in this study is based on the expected variations between animals and is comparable to many previous reports using similar techniques. Sample size of each experiments can be found in the Figure legends.<br>Chen, Z. K. et al. A cluster of mesopontine GABAergic neurons suppresses REM sleep and curbs cataplexy. Cell Discov. 8, 115 (2022).<br>Chen, Z. K. et al. Whole-brain neural connectivity to lateral pontine tegmentum GABAergic neurons in mice. Front. Neurosci. 13, 375 (2019).<br>Wander, C. M. et al. Compensatory remodeling of a septo-hippocampal GABAergic network in the triple transgenic Alzheimer's mouse model. J. Transl. Med. 21, 258 (2023).<br>Huang, Z. L. et al. Adenosine A2A, but not A1, receptors mediate the arousal effect of caffeine. Nat. Neurosci. 8, 858–859 (2005).
Data exclusions	Data from animals were excluded based on histological criteria that included injection sites,virus expression and optical fiber placement. Only animals with injection sites/virus expression/optical fiber placement in the region of interest were included, based on our previous reports.
Replication	All experiments were replicated 2-3 times, and all attempts at replication were successful.
Randomization	The experiments were not randomized. Animal were allocated into experimental groups by matched gender, age, weight, etc.
Blinding	Investigators were blinded to the experimental groups until all data had been collected and analyzed.

Reporting for specific materials, systems and methods

We require information from authors about some types of materials, experimental systems and methods used in many studies. Here, indicate whether each material, system or method listed is relevant to your study. If you are not sure if a list item applies to your research, read the appropriate section before selecting a response.

Materials & experimental systems

n/a	Involved in the study	
☐	☒	Antibodies
☒	☐	Eukaryotic cell lines
☒	☐	Palaeontology and archaeology
☐	☒	Animals and other organisms
☒	☐	Clinical data
☒	☐	Dual use research of concern
☒	☐	Plants

Methods

n/a	Involved in the study	
☒	☐	ChIP-seq
☒	☐	Flow cytometry
☒	☐	MRI-based neuroimaging

Antibodies

Antibodies used	Anti-Goat GFAP Santa Cruz Biotechnology Cat# sc-6170,<br>Anti-Rabbit Iba1 Fujifilm Wako Cat# 019-19741,<br>Anti-Chichen Neuron Specific Enolase Cat# AB9698,<br>Neurotrace 435/455 Blue Fluorescent Nissl stain Invitrogen Cat# N21479,<br>Alexa Fluor 647 Donkey anti-Chicken Invitrogen Cat # A78952,<br>Alexa Fluor 568 Donkey anti-Chicken Invitrogen Cat # A78950,<br>Alexa Fluor 647 Donkey anti-Rabbit Invitrogen Cat # A32795,<br>Alexa Fluor 568 Donkey anti-goat Invitrogen Cat # A11057,<br>Alexa Fluor 488 Donkey anti-goat Invitrogen Cat # A11055,
Validation	All of the antibody calidation information can be found online.<br>Anti-Goat GFAP Santa Cruz Biotechnology: https://www.scbt.com/p/gfap-antibody-2e1;<br>Anti-Rabbit Iba1 https://labchem-wako.fujifilm.com/us/product/detail/W01W0101-1974.html;<br>Anti-Chichen Neuron Specific Enolase https://www.emdmillipore.com/US/en/product/Anti-Neuron-Specific-Enolase-NSE-Antibody,MM_NF-AB9698;<br>Neurotrace 435/455 Blue Fluorescent Nissl stain https://www.thermofisher.com/order/catalog/product/N21479?gclid=Cj0KCQjwqP2pBhDMARIsAJQ0CzqjKEhCa445hzFxxhfW6Teg_DTODlflq5xKL5wNIfTBYfmoYWARuZsaAvWBEALw_wcB&ef_id=Cj0KCQjwqP2pBhDMARIsAJQ0CzqjKEhCa445hzFxxhfW6Teg_DTODlflq5xKL5wNIfTBYfmoYWARuZsaAvWBEALw_wcB:G:s&s_kwcid=AL!3652!3!447292198730!!!g!!!10506731179!109642167491&cid=bid_pca_iva_r01_co_cp1359_pjt0000_bid00000_0se_gaw_dy_pur_con&gad_source=1

Animals and other research organisms

Policy information about studies involving animals; ARRIVE guidelines recommended for reporting animal research, and Sex and Gender in Research

Laboratory animals	Mice were used from 8-16 weeks, mouse is listed below: C57BL/6J (Strain #:000664) mice.<br>Mice had access to food and water ad libitum and were maintained at constant temperature (22–24 °C), humidity (40–60%), and 12 h light/dark cycle.
Wild animals	No wild animals were used in the study.
Reporting on sex	Both male and female B6 mice were used.
Field-collected samples	No field collected samples were used in the study.
Ethics oversight	All procedures were conducted in accordance with the NIH Guide for the Care and Use of Laboratory Animals and with the approval of the Institutional Animal Care and Use Committee at the University of North Carolina at Chapel Hill (UNC).

Note that full information on the approval of the study protocol must also be provided in the manuscript.

Plants

Seed stocks	N/A
Novel plant genotypes	N/A
Authentication	N/A
